# Noninvasive prediction of response to cancer therapy using promoter profiling of circulating cell‐free DNA

**DOI:** 10.1002/ctm2.174

**Published:** 2020-09-20

**Authors:** Zhi‐Wei Guo, Wei‐Wei Xiao, Xue‐Xi Yang, Xu Yang, Geng‐Xi Cai, Xiao‐Jing Wang, Bo‐Wei Han, Kun Li, Xiang‐Ming Zhai, Fen‐Xia Li, Li‐Min Huang, Ying‐Song Wu, Yuan‐Hong Gao

**Affiliations:** ^1^ Institute of Antibody Engineering School of Laboratory Medicine and Biotechnology Southern Medical University Guangzhou China; ^2^ Department of Radiation Oncology Sun Yat‐sen University Cancer Center Guangzhou China; ^3^ State Key Laboratory of Oncology in South China Collaborative Innovation Center for Cancer Medicine Guangzhou China; ^4^ Department of Breast Surgery The First People's Hospital of Foshan Foshan China; ^5^ Anhui Clinical and Preclinical Key Laboratory of Respiratory Disease, The Center of Molecular Diagnosis, The Department of Respiratory and Critical Care Medicine The First Affiliated Hospital of Bengbu Medical College Bengbu China; ^6^ Department of Obstetrics and Gynecology Nanfang Hospital Southern Medical University Guangzhou China; ^7^ XGene Co., Ltd Guangzhou China

Dear Editor,

Neoadjuvant radiotherapy and concomitant fluorouracil‐based chemoradiotherapy (CRT) followed by total mesorectal excision is the conventional treatment for locally advanced rectal cancer (LARC).^1^ However, there are individual differences in the sensitivity of cancer patients to cancer therapies.^2^ The effectiveness prediction before treatment would assist clinical decisions, effectively avoid indiscriminately using drugs, reduce side effects, and improve the curative effect and quality of life.^3^ Therefore, it is important to develop a novel noninvasive methodology to predict the effectiveness of cancer therapy before cancer treatment, which would enable timely interventions and provide a more individualized approach for better treatment outcomes.

Cell‐free DNA (cfDNA) is mainly derived from tumor and hematopoietic cells in cancer patients and it can reflect the characteristics of its tissue of origin.^4^, ^5^ For instance, the promoter coverage of cfDNA could be used to infer the expression status of tumor tissue.^5^ As tumor expression status is closely related to patient's responses to cancer therapy,^6^ we hypothesized that the promoter profiling of cfDNA could be used for pathologic complete response (pCR) prediction after neoadjuvant CRT. In this study, we first compared the local chromatin changes of cfDNA between the pCR and non‐pCR groups of LARC patients. We further evaluated the potentials of promoter profiling of cfDNA for predicting the effectiveness of cancer therapy by developing classifiers for distinguishing pCR and non‐pCR patients (Figure 1A).

**FIGURE 1:**
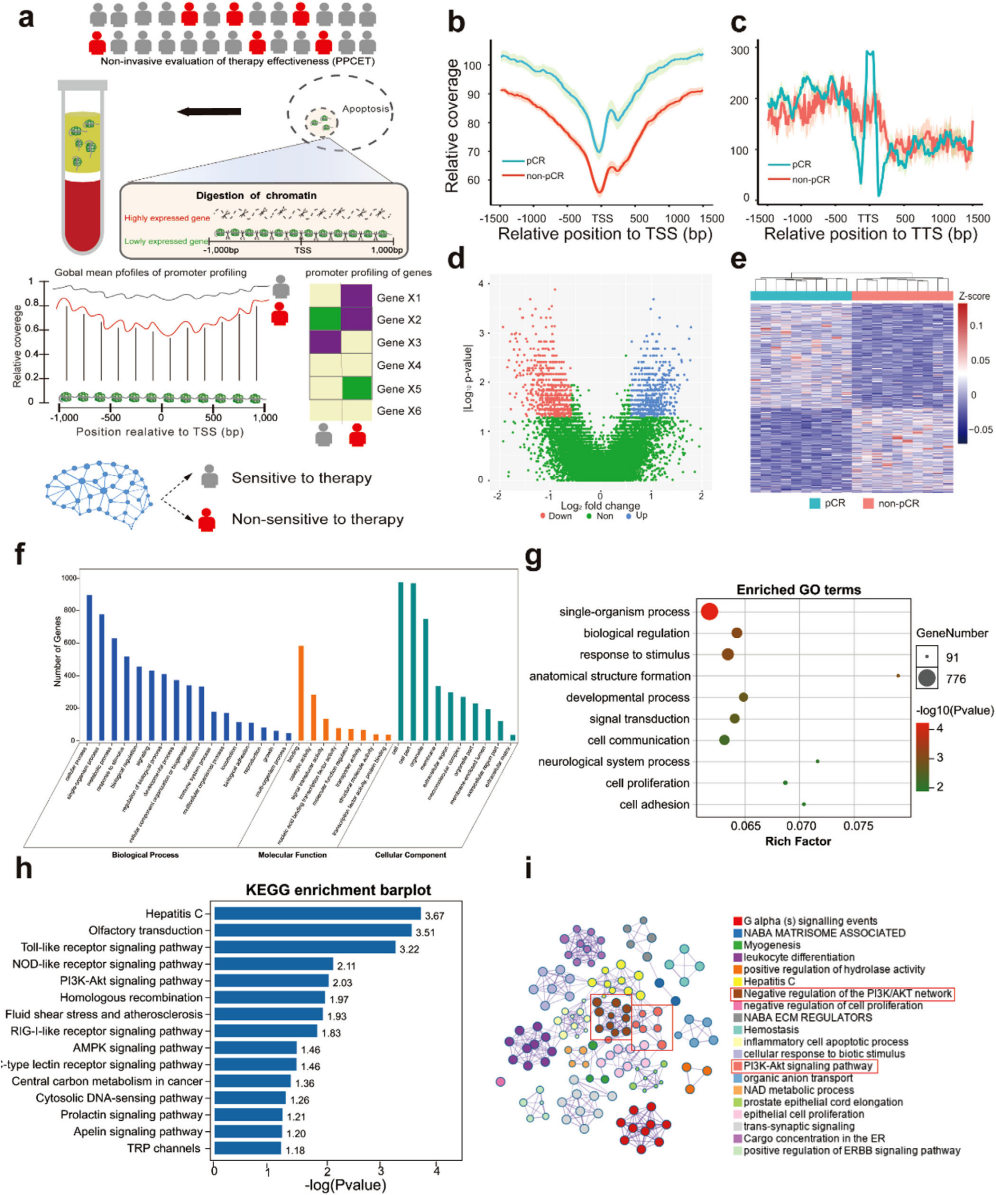
Promoter profiling shows potentials for assessing the effectiveness of cancer therapy. A, Schematic overview of PPCET. In cancer, the plasma cell‐free DNA (cfDNA) is primarily derived from tumor and hematopoietic cells. Exposed DNA not bound to a nucleosome is digested, whereas nucleosome‐bound DNA escapes digestion and enters into circulation. Therefore, cfDNA comprises a nucleosome footprint that carries information of its tissue of origin and could reflect its gene expression pattern. As the effectiveness of cancer therapy is closely related to expression status of tumor and hematopoietic cells, which highly expressed genes in original tissue may show lower DNA coverage than lowly expressed genes in cfDNA, we hypothesized that the read coverage of cfDNA at gene promoters could be used to develop classifiers for predicting the patient responses to cancer therapy. To show greater differences, all nucleosome in the promoter regions (−1 kB to +1 kB around the transcription start site [TSS]) of highly expressed genes are depleted; however, the nucleosome‐depleted region is usually found within the nucleosome upstream of the TSS. **B,** Average cell‐free DNA signals at TSS. **C,** Average cell‐free DNA signals at transcriptional terminal sites (TTS). **D,** Volcano plots of gene transcripts with differential read coverages at promoter (fold change ≥ 1.5 and false discovery rate [FDR] ≤ 0.05) between pCR and non‐pCR patients. **E,** Heat map of the *z*‐scores of promoters with differential read coverages. TSS, transcriptional start sites. **F,** Gene ontology (GO) annotation of genes with differential promoter coverage. **G,** Top 10 enriched GO biological processes. **H,** Top 10 enriched Kyoto Encyclopedia of Genes and Genomes (KEGG) pathways. **I,** Network of biological processes of genes with differential promoter coverage. pCR, partial clinical response; non‐pCR, nonpartial clinical response

By comparing the local cfDNA signal between 10 pCR and 10 non‐pCR patients, we observed a related loss of cfDNA signals in the mean coverage of transcriptional start site (TSS) in LARC patients with non‐pCR (Figure 1B, *P*‐value = 3.4 × 10^−21^, Wilcoxon rank‐sum test). But the cfDNA signals around transcriptional terminal site (TTS) did not show significant difference (Figure 1C, *P*‐value = 1, Wilcoxon rank‐sum test). Therefore, we further compared their promoter profiling for each TSS, we identified 1371 genes with differential promoter coverage (Figure 1D and Table S1, fold change ≥ 1.5 and false discovery rate [FDR] ≤ 0.05, Wilcoxon rank‐sum test). The results of unsupervised clustering analysis revealed that the promoter profiling between the pCR and non‐pCR patients showed distinctive coverage patterns (Figure 2D).

**FIGURE 2:**
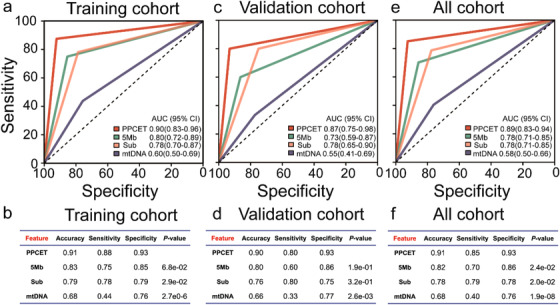
Prediction of pCR patients using PPCET. Receiver operating characteristic (ROC) curves for the training cohort **(A)**, validation cohort **(C),** and all cohorts **(E)**. Performance of classifiers for the training cohort **(B)**, validation cohort **(D),** and all cohorts **(F)**. In total, 194 LARC patients with neoadjuvant therapy were used to develop classifiers to predict pCR patients. The classifier PPCET with the largest area under curve (AUC) was identified (AUC = 0.89 [0.83‐0.94]). Machine learning analysis of the variables of the global nucleosome, including fragment profiles of 5 Mb windows (5Mb), subcompartments of genome (Sub), and mitochondrial DNA copy number (mtDNA), was implemented

Gene Ontology (GO) and Kyoto Encyclopedia of Genes and Genomes (KEGG) pathway enrichment analysis was implemented and visualized using Metascape^7^ and OmicShare tools (www.omicshare.com/tools). The results showed that the genes with differential coverage were associated with the patient's response to cancer therapy (Figure 1F‐I). For example, the genes with differential promoter coverage were enriched in multiple pathways, such as Toll‐like receptor (TLR), NOD‐like receptor (NLR), PI3K‐Akt, and AMPK signaling pathway (Figure 1H). Previous studies have shown these pathways were closely related to the patient's response to cancer therapy.^8^, ^9^ For instance, TLR pathway can increase the sensitivity to chemotherapy.^8^ NLR pathway can affect the sensitivity to radiotherapy or chemotherapy.^9^ These results may indicate that promoter profiling may be useful for predicting the effectiveness of cancer therapy before treatment.

To further validate the potential of promoter profiling for pCR prediction after neoadjuvant CRT, we detected the whole‐genome sequencing (WGS) of cfDNA derived from 194 LARC patients with pCR (n = 47) and non‐pCR patients (n = 147) collected before therapy (Figure S1 and Table S2). According to the collection time of plasma samples, the participants were split into training and validation cohorts. Using the genes with differential promoter coverage identified in the discovery stage, we implemented a SVM machine learning model to predict the response to cancer therapy, and estimated the performance characteristics of this approach by leave one out cross‐validation (LOOCV) cross‐validation (Supporting Information Materials). We used ROC analysis to evaluate the area under curve (AUC), sensitivity, specificity, and accuracy of the promoter profiling classifiers (Figure 2). Among all combinations, a nine‐gene combination achieved high performance (AUC = 0.90 [95% confidence interval 0.83‐0.96] and accuracy = 0.91) in the training cohort after LOOCV, displaying the largest AUC (Figure 2A and B). Then, this classifier, named PPCET, was subsequently studied and validated in the validation cohort with an AUC of 0.87 (0.75‐0.98, Figure 2C and D). The genes in PPCET were *MRGPRX2*, *OR10A2*, *GOLT1A*, *DLEU7*, *KLH1*, *MED25*, *NDUFA7*, *SUMF2*, and *HDHD3* (Table S3).

In all cohorts, the PPCET had an AUC of 0.89 (0.83‐0.94) to discriminate individuals with pCR from non‐pCR patients with a sensitivity of 0.85 and specificity of 0.93 (Figure 2E and F). Previous studies have shown that the global chromatin between different types of cancer was significantly different,^10^ which indicated that it could be used for predicting patients’ response to cancer therapy. Compared with other global chromatin variables (Supporting Information Materials), the AUC for PPCET was significantly greater than those for the classifiers based on fragment profiles of 5 Mb windows (0.78 [0.71‐0.85], *P‐*value = 2.4 × 10^−02^), subcompartments (0.78 [0.71‐0.85], *P‐*value = 2.0 × 10^−02^), and mitochondrial DNA copy number (0.58 [0.50‐0.66], *P‐*value = 1.9 × 10^−08^, Figure 2E and F). These results may suggest that the classifier based on promoter profiling could effectively predict the effectiveness of cancer therapy.

PPCET based on circulating cfDNA is a noninvasive method and could avoid heterogeneity of tumor detection. In addition, PPCET requires the plasma samples collected before treatment to predict patient response after therapy, which may enable timely interventions and provide a more individualized method for better treatment outcomes. There are also some limitations to our study: The patients may show a different degree of sensitivity to cancer therapy in the clinic, but we only separated the patients into pCR and non‐pCR groups because of a limitation of sample size.

In summary, our data suggest that PPCET is a noninvasive promising method based on low‐coverage DNA sequencing for predicting the response of cancer patients to cancer therapy before treatment. PPCET may help to prevent indiscriminate use of drugs, reduce toxicity and side effects, and improve the curative effect and quality of life.

## CONFLICT OF INTEREST

The authors declare no conflict of interest.

## ETHICS APPROVAL AND CONSENT TO PARTICIPATE

All plasma samples were obtained under Institutional Review Board approved protocols with informed consent from all participants for research use.

## AUTHOR CONTRIBUTIONS

Yuan‐Hong Gao, Xue‐Xi Yang and Ying‐Song Wu designed and supervised the study. Zhi‐Wei Guo, Xu Yang, Bo‐Wei Han, and Kun Li, analyzed and interpreted the data and prepared the manuscript. Yuan‐Hong Gao, Wei‐Wei Xiao, Geng‐Xi Cai, and Xiao‐Jing Wang provided samples and interpreted clinical data. Xiang‐Ming Zhai, Fen‐Xia Li, and Li‐Min Huang performed whole genome sequencing.

## FUNDING INFORMATION

National Natural Science Foundation of China; Grant Numbers: 81802435, 81672987 and 81872416; China Postdoctoral Science Foundation; grant Numbers: 2019T120742 and 2016M602486; Natural Science Foundation of Guangdong Province; Grant Numbers: 2018A030313286 and 2015B020233009; Guangzhou Science and Technology Program key projects; Grant Numbers: 201604020104 and 201803040009.

## Supporting information

Supporting Information

## Data Availability

The aligned reads of the merged samples reported in this paper have been deposited at the database of NODE (https://www.biosino.org/node, project ID: OEP000648).
