# Supplementary material for: Noninvasive prediction of response to cancer therapy using promoter profiling of circulating cell‐free DNA
Source: Clin Transl Med. 2020 Sep 20;10(5):e174. doi: 10.1002/ctm2.174 (PMC7507006; doi:10.1002/ctm2.174)
Supplement: Supplementary file 1 — Supporting Information [file CTM2-10-e174-s001.docx]

**Supplementary Materials**

**Supplementary Method**

**Participants and study design**

In total, we prospectively collected plasma samples of cancer patients before therapy. According to the responses to cancer therapy, the participants were divided into two groups: LARC patients with pCR (n=47) or non-pCR (n=147) after neoadjuvant chemoradiotherapy collected from Sun Yat-sen University Cancer Center (SYSUCC). All plasma samples were obtained under institutional review board of SYSUCC approved protocols with written informed consent from all participants for research use. This study consisted of three stage, including discovery, training and validation stages to develop classifier for predicting the effectiveness of cancer therapy (Supplementary Figure 1). Plasma samples used in the discovery stage were prospectively collected between Jan 2015 and Dec 2015. Plasma samples used in the training and validation stages were prospectively collected between Jan 2016 and Jan 2019.

All of the patients were histopathologically confirmed and none of them had received anti-tumor therapy before sampling. LARC patients received neoadjuvant chemoradiotherapy, which received mFolfox/Xelox and radiotherapy. According to Response Evaluation Criteria in Solid Tumors criteria (RECIST) [^1^](#_ENREF_1), the patients were classified as pCR/non-pCR groups. Clinical data of all patients in more detail was showed in Supplementary Table 2.

**Sample preparation and sequencing of cfDNA**

Plasma cfDNA was extracted using the QIAamp DNA Blood Mini kit (Qiagen, Hilden, German) by following the manufacturer’s protocol. DNA concentration and integration were measured using a Qubit fluorimeter (ThermoFisher Scientific, Waltham, MA, USA) and an Agilent Bioanalyzer 2100 (Agilent Technologies, Santa Clara, CA, USA). DNA was eluted in 50 µL AE buffer and stored at −20°C. Libraries were prepared according to the manufacturer’s instructions and then sequenced on an Ion Proton System on a P1 chip (Life Technologies, Carlsbad, CA, USA). After removal of sequencing reads with low quality, sequencing reads were aligned to the human reference genome (hg19) using TMAP (ver.5.0.13), and then the PCR duplicated were removed using the SAMtools rmdup function (ver. 0.1.18) [^2^](#_ENREF_2). In general, each sample was sequenced at a mean depth of 0.3🞨 average coverage.

**Global and local chromatin changes of cfDNA**

In the discovery stage, we first compared the global and local chromatin changes between sensitive groups and non-sensitive groups in LARC (pCR=10 and non-pCR=10, Supplementary Figure 1).

To identify the global nucleosome changes, we compared the fragment profiles at 5-megabase (Mb) windows, sub-compartments and the copy number of the mitochondrial genome between two groups of patients in each cancer type. To calculate the fragment profiles at 5-Mb windows and sub-compartments, we first tiled the hg19 autosomes into adjacent, non-overlapping 100-kilobase (kb) bins and filtered the bins more than 10% of regions overlapping with the Duke blacklisted regions (http://hgdownload.cse.ucsc.edu/goldenpath/hg19/encodeDCC/wgEncodeMapability). To account for the biases in coverage attributable to GC content of the genome, we used locally weighted scatterplot smoothing regression (LOWESS) with a span setting of 0.75 for each sample (Supplementary Figure 2). We returned the residuals to the original scale by adding back the mean coverage of each sample. Then, the coverage of each 5-Mb window was calculated by adding up the GC-adjusted coverage values of the 100-kb bins within it. For the fragment profiles of sub-compartments, the human genome was first annotated by the Hi‐C data of GM12878 [^3^](#_ENREF_3), and then the coverage of each sub-compartment was calculated by adding up the GC-adjusted coverage of the 100-kb bins. For the copy number of the mitochondrial genome, the number of mapped sequences to the mitochondrial genome was divided by the mapped sequences to the whole genome. Finally, the fragment profiles of 5-Mb windows, sub-compartments and the copy number of the mitochondrial genome were then standardized based on z-scores.

To compare local chromatin changes, gene information was first downloaded from RefSeq of the University of California Santa Cruz (UCSC) [^4^](#_ENREF_4). DANPOS was used to assess the cfDNA signals at each transcriptional start site (TSS) and transcriptional terminal site (TTS) [^5^](#_ENREF_5). To identify local chromatin changes of each TSS, cfDNA coverage around TSSs (−1,000 bp to +1,000 bp of TSS, defined as promoter profiling) was extracted using bedtools (ver. 2.17.0) [^6^](#_ENREF_6). Then the normalized value of promoter profiling was calculated using the following formula:

$$Promoter profiling=\frac{cfDNA coverage around TSS}{Totally mapped reads}$$

**Models for predicting the responses to neoadjuvant chemoradiotherapy**

To identify the optimal variables for predicting the effectiveness of cancer therapy among the global and local chromatin features, we performed WGS of cfDNA derived from 194 LARC patients with pCR (n=47) or non-pCR (n=147) (Supplementary Figure 1). According to the collection time of plasma, the samples of each group were divided into training and validation cohorts with a ratio of 7:3. In the training stage, we developed classifiers with SVM models to distinguish pCR from non-pCR patients using global (fragment profiles at 5-Mb windows, sub-compartments and mitochondrial DNA copy number), local chromatin variables (promoter profiling) identified in the discovery stage and clinical features. As a considerable number of studies have reported that discrete data may improve classifier performance[^7^](#_ENREF_7), the continuous variable was first discretized according to the optimal cut-off point before classifier construction. The optimal cut-off point of each variable was defined as the maximum value of (sensitivity + specificity)/2 in the training cohort. Then the continuous value was set to one when it was larger than the corresponding optimal cut-off in each subject; otherwise, it was set to zero (Supplementary Table 3). By using the stepwise method, the optimal classifier with the largest AUC was selected. To estimate the prediction error of this approach, we used the leave one out cross-validation (LOOCV) method. Briefly, each subject in the training cohort was withheld in turn, and the remaining subjects were submitted to train the model. As there were 135 samples in the training cohort, this procedure was repeated 135 times. Then, the performance of the optimal classifier was further validated in the validation cohort.

**Statistical analysis**

We used Wilcoxon rank sum test for analyses that compared two groups. The raw *P*-values were then adjusted to the false discovery rate (FDR) using the Benjamini-Hochberg procedure. The variables with fold change ≥ 1.5 and FDR < 0.05 were considered to be statistically significant. Hierarchical clustering was applied to the coverage data, using the average‐linkage clustering algorithms in Cluster (ver. 3.0). Heat maps were plotted using the pheatmap package in R software (version 3.0.1). SVM were implemented using the e1071 package using linear kernel with default setting. We plotted the receiver operating characteristic (ROC) curve and evaluated the difference of area under the ROC curve (AUC) using the pROC package[^8^](#_ENREF_8) of R. GO and KEGG pathway enrichment analysis was implemented and visualized using Metascape [^9^](#_ENREF_9) and OmicShare tools ([www.omicshare.com/tools](http://www.omicshare.com/tools)). The functional relevance between the top enriched pathways and patient responses to cancer therapy were annotated by searching the literature.

**Supplementary Figures and Tables**

| **Supplementary Figures** |
| --- |
| Supplementary Figure 1. Study design. |
| Supplementary Figure 2. Genome-wide GC correction of cfDNA fragment profiles. |
| Supplementary Figure 3. Global nucleosome changes between pCR and non-pCR LARC patients after neoadjuvant chemoradiotherapy |
| **Supplementary Tables** |
| Supplementary Table 1. Genes with differential promoter coverage between sensitive and non-sensitive groups |
| Supplementary Table 2. Clinical characteristics of locally advanced rectal cancer |
| Supplementary Table 3. Thresholds of promoter profiling for each gene in PPCET |

**Supplementary Figures**


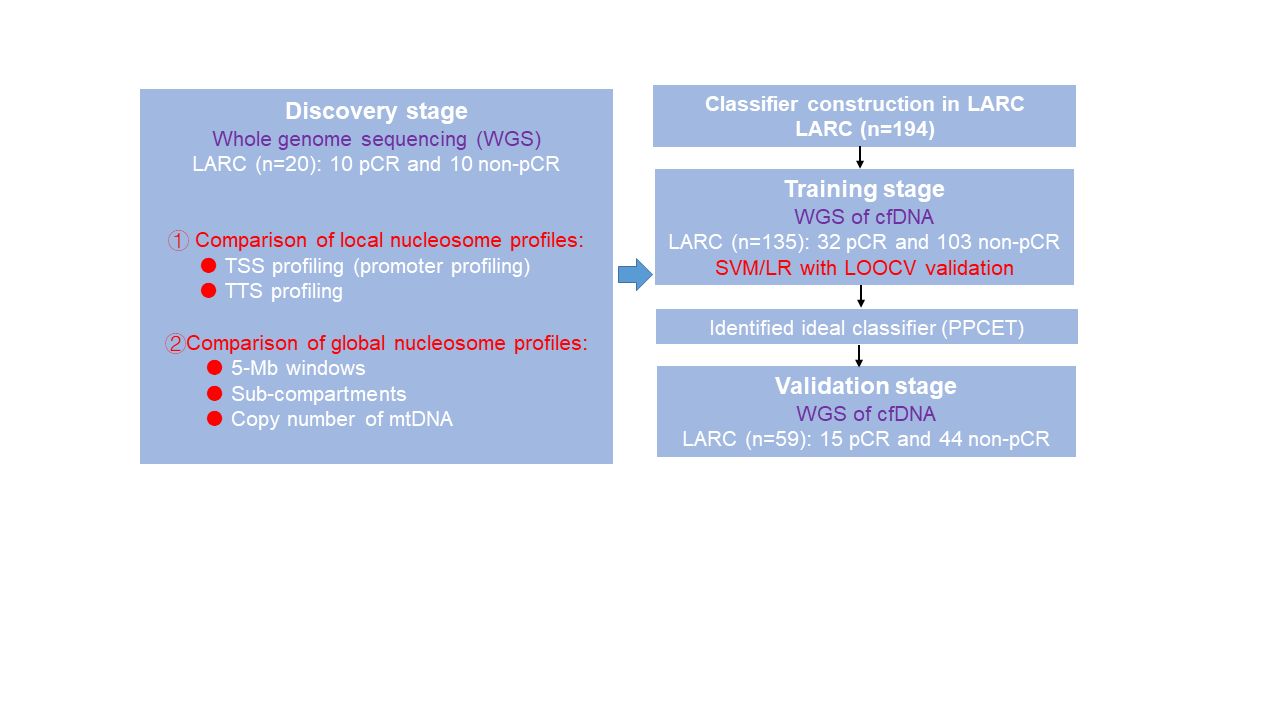


**Supplementary Figure 1. Study design**. According to Response Evaluation Criteria in Solid Tumors criteria (RECIST), the patients were defined as pCR and non-pCR. LARC=locally advanced rectal cancer received mFolfox/Xelox and radiotherapy. More details about cancer patients were showed in method section. TSS=transcriptional start site. TTS=transcriptional terminal site. pCR=pathological complete response. SVM=support vector machine. LR=logistic regression. LOOCV=leave one out cross validation. PPCET=promoter profiling of cfDNA for effectiveness evaluation before cancer therapy.

**
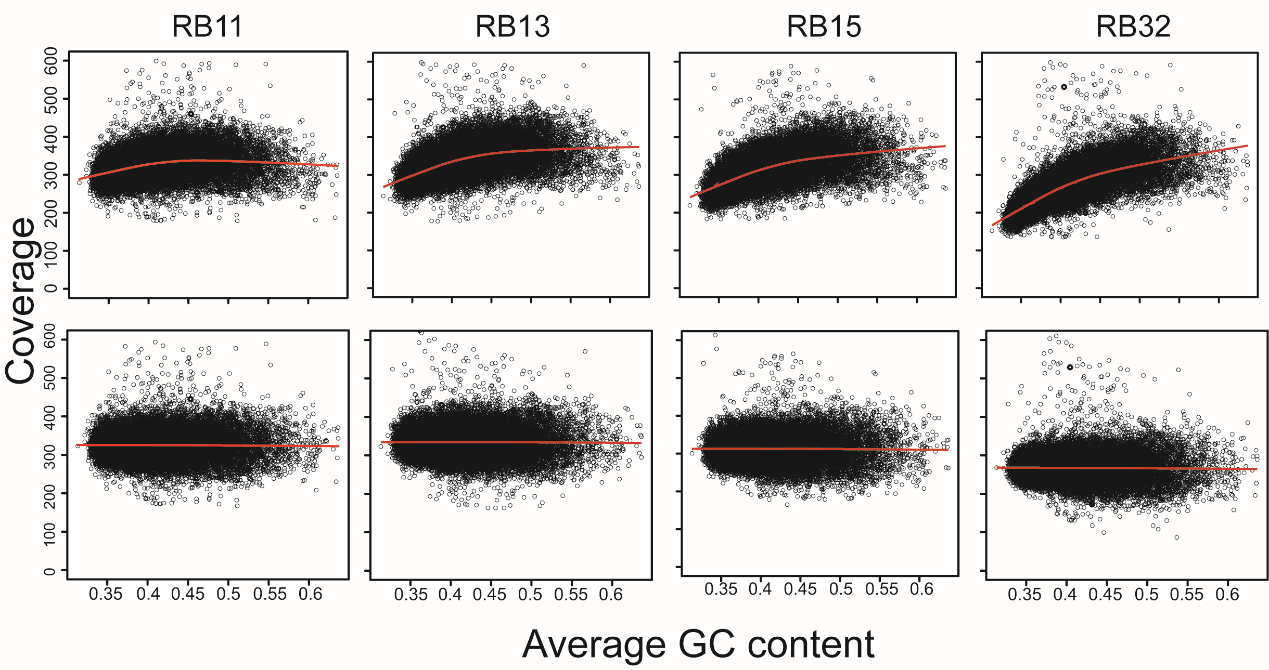
**

**Supplementary Figure 2. Genome-wide GC correction of cfDNA fragment profiles**. To assess and control for the GC effects on sequencing coverage, we calculated coverage in non-overlapping 100-kb genomic windows across the autosomes. The raw coverage (top row) for four randomly selected locally advanced rectal cancer patients (LARC; RB11, RB13, RB15, and RB32). Local weighted regression (LOWESS) smoothing of raw coverage for advanced rectal cancer patients (bottom row).


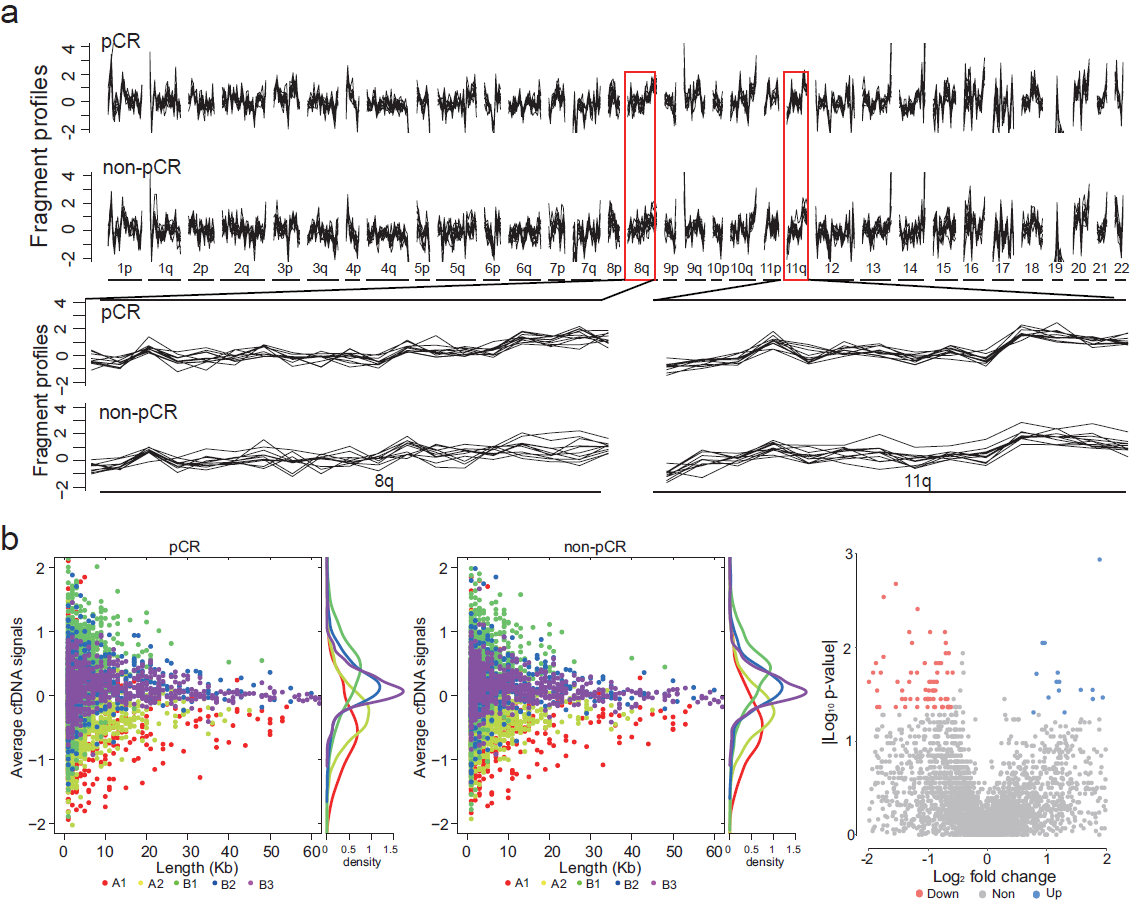


**Supplementary Figure 3. Global nucleosome changes between pCR and non-pCR LARC patients after neoadjuvant chemoradiotherapy. a,** Genome-wide cfDNA fragmentation profiles of the WGS are shown in 5-Mb bins for pCR and non-pCR patients. The red box showed the detailed cfDNA fragmentation profiles from chromosome 8q and 11q at 5-Mb resolution for pCR and non-pCR patients. **b,** genome-wide cell‐free DNA fragment profiles in different sub-compartments for LARC patients with pCR and non-pCR patients. The distribution of sub-compartments with differential read coverages. Volcano plots of sub-compartments with differential read coverages (fold change > 1.5 and false discovery rate [FDR] < 0.05) between pCR and non-pCR patients. pCR=partial clinical response. Non-pCR=non-partial clinical response. The sub-compartment of the human genome was annotated by the Hi‐C data of GM12878. A1 and A2 regions are gene-enriched regions. B1 consists of facultative heterochromatic regions. B2 is enriched at the nuclear lamina and at nucleolus‐associated domains (NADs). B3 is also enriched at the nuclear lamina but not at NADs. Down (red), non (grey) and up (blue) means sub-compartments with downregulated, non-significantly different and upregulated coverage.

**Supplementary Tables**

**Supplementary Table 1. Genes with differential promoter coverage between sensitive and non-sensitive groups**

| Type | RefSeq | Position | Fold change | FDR |
| --- | --- | --- | --- | --- |
| LARC | NM_001080526 | chr8.82372758.82374758 | 0.317 | 0.015 |
| LARC | NM_001004731 | chr14.21623184.21625184 | 0.52 | 0.015 |
| LARC | NM_001005484 | chr1.68090.70090 | 0.365 | 0.026 |
| LARC | NM_001242329 | chr4.9335383.9337383 | 1.744 | 0.026 |
| LARC | NM_001005469 | chr11.58169882.58171882 | 0.248 | 0.033 |
| LARC | NM_001291957 | chr5.19885381.19887381 | 0.33 | 0.033 |
| LARC | NM_001270 | chr5.98261238.98263238 | 0.34 | 0.033 |
| LARC | NM_007281 | chr4.174319397.174321397 | 0.344 | 0.033 |
| LARC | NM_002302 | chr5.135289723.135291723 | 0.38 | 0.033 |
| LARC | NM_001004757 | chr11.5442340.5444340 | 0.39 | 0.033 |
| LARC | NM_001004733 | chr11.58206646.58208646 | 0.409 | 0.033 |
| LARC | NM_001005180 | chr11.5756677.5758677 | 0.44 | 0.033 |
| LARC | NM_001348271 | chr14.20469220.20471220 | 0.473 | 0.033 |
| LARC | NM_024721 | chr8.77592514.77594514 | 0.492 | 0.033 |
| LARC | NM_001004473 | chr1.158434351.158436351 | 0.501 | 0.033 |
| LARC | NM_001081552 | chr8.7704401.7706401 | 0.511 | 0.033 |
| LARC | NM_001291283 | chr1.248902151.248904151 | 0.534 | 0.033 |
| LARC | NM_001204267 | chr4.46994522.46996522 | 1.98 | 0.033 |
| LARC | NM_182791 | chr15.74609881.74611881 | 2.06 | 0.033 |
| LARC | NM_032423 | chr19.52900120.52902120 | 2.236 | 0.033 |
| LARC | NM_001321233 | chr2.17710681.17712681 | 0.238 | 0.034 |
| LARC | NM_001258380 | chr3.27497245.27499245 | 0.245 | 0.034 |
| LARC | NM_001271539 | chr20.13970265.13972265 | 0.262 | 0.034 |
| LARC | NM_004950 | chr12.91397803.91399803 | 0.288 | 0.034 |
| LARC | NM_018908 | chr5.140200360.140202360 | 0.295 | 0.034 |
| LARC | NM_080874 | chr4.177189373.177191373 | 0.308 | 0.034 |
| LARC | NM_001290263 | chr2.225810782.225812782 | 0.312 | 0.034 |
| LARC | NM_020432 | chr7.77468446.77470446 | 0.313 | 0.034 |
| LARC | NM_001004756 | chr11.5409606.5411606 | 0.317 | 0.034 |
| LARC | NM_001277185 | chr6.130536030.130538030 | 0.329 | 0.034 |
| LARC | NM_032681 | chr11.55649772.55651772 | 0.359 | 0.034 |
| LARC | NM_001161528 | chr7.91793590.91795590 | 0.365 | 0.034 |
| LARC | NM_015230 | chr4.36244979.36246979 | 0.395 | 0.034 |
| LARC | NM_006609 | chr2.128099805.128101805 | 0.411 | 0.034 |
| LARC | NM_000845 | chr7.126882569.126884569 | 0.429 | 0.034 |
| LARC | NM_001012418 | chr6.2750200.2752200 | 0.441 | 0.034 |
| LARC | NM_001204415 | chr1.32042745.32044745 | 0.46 | 0.034 |
| LARC | NM_001080534 | chr15.54303844.54305844 | 0.462 | 0.034 |
| LARC | NM_001321770 | chr2.108993366.108995366 | 0.471 | 0.034 |
| LARC | NM_001302644 | chr11.111174773.111176773 | 0.473 | 0.034 |
| LARC | NM_001004460 | chr11.6889985.6891985 | 0.501 | 0.034 |
| LARC | NM_001321896 | chr7.18328714.18330714 | 0.505 | 0.034 |
| LARC | NM_001005173 | chr11.6007215.6009215 | 0.511 | 0.034 |
| LARC | NM_001348164 | chr19.12202077.12204077 | 0.511 | 0.034 |
| LARC | NM_001353802 | chr9.6247480.6249480 | 0.515 | 0.034 |
| LARC | NM_173538 | chr8.87877675.87879675 | 0.517 | 0.034 |
| LARC | NM_001161522 | chr4.166130170.166132170 | 0.523 | 0.034 |
| LARC | NM_016512 | chr8.7320192.7322192 | 0.53 | 0.034 |
| LARC | NM_181519 | chr10.46969601.46971601 | 1.528 | 0.034 |
| LARC | NM_001037553 | chr21.45344278.45346278 | 1.819 | 0.034 |
| LARC | NM_001034914 | chr2.96011767.96013767 | 1.874 | 0.034 |
| LARC | NM_001286725 | chr13.70681625.70683625 | 1.897 | 0.034 |
| LARC | NM_153018 | chr17.4980753.4982753 | 2.018 | 0.034 |
| LARC | NM_001164104 | chr22.50527434.50529434 | 2.039 | 0.034 |
| LARC | NM_001318832 | chr16.2097202.2099202 | 2.133 | 0.034 |
| LARC | NM_207361 | chr13.39260172.39262172 | 2.163 | 0.034 |
| LARC | NM_007059 | chr19.47986521.47988521 | 2.225 | 0.034 |
| LARC | NM_001278633 | chr5.1886293.1888293 | 2.855 | 0.034 |
| LARC | NM_001258026 | chr12.6976277.6978277 | 3.355 | 0.034 |
| LARC | NM_139284 | chr19.35625178.35627178 | 3.44 | 0.034 |
| LARC | NM_001024674 | chr14.74550655.74552655 | 0.304 | 0.035 |
| LARC | NM_001282598 | chr2.79749308.79751308 | 0.328 | 0.035 |
| LARC | NM_001134745 | chr2.77748559.77750559 | 0.381 | 0.035 |
| LARC | NM_001004482 | chr9.107360694.107362694 | 0.399 | 0.035 |
| LARC | NM_018324 | chr10.15084894.15086894 | 0.424 | 0.035 |
| LARC | NM_181622 | chr21.31797244.31799244 | 0.45 | 0.035 |
| LARC | NM_001005468 | chr11.124252239.124254239 | 0.452 | 0.035 |
| LARC | NM_001994 | chr1.197035397.197037397 | 0.467 | 0.035 |
| LARC | NM_001288774 | chr12.18413473.18415473 | 0.483 | 0.035 |
| LARC | NM_001136555 | chr2.171922483.171924483 | 0.491 | 0.035 |
| LARC | NM_001005287 | chr7.143928936.143930936 | 0.517 | 0.035 |
| LARC | NM_054030 | chr11.19081228.19083228 | 0.523 | 0.035 |
| LARC | NM_014913 | chr18.77865914.77867914 | 1.733 | 0.035 |
| LARC | NM_001172501 | chr16.55688541.55690541 | 1.886 | 0.035 |
| LARC | NM_001207038 | chr6.36354568.36356568 | 2.05 | 0.035 |
| LARC | NM_001114382 | chr16.2096895.2098895 | 2.071 | 0.035 |
| LARC | NM_001083 | chr4.120548981.120550981 | 2.074 | 0.035 |
| LARC | NM_001199303 | chr12.70636493.70638493 | 2.131 | 0.035 |
| LARC | NM_001001480 | chr11.1650032.1652032 | 2.162 | 0.035 |
| LARC | NM_001164105 | chr22.50527684.50529684 | 2.199 | 0.035 |
| LARC | NM_001030005 | chr15.75117950.75119950 | 2.407 | 0.035 |
| LARC | NM_018712 | chr11.107460816.107462816 | 2.68 | 0.035 |
| LARC | NM_205858 | chr15.85200802.85202802 | 2.745 | 0.035 |
| LARC | NM_001304944 | chr12.49739699.49741699 | 4.231 | 0.035 |
| LARC | NM_001487 | chr12.56108817.56110817 | 5.234 | 0.035 |
| LARC | NM_002947 | chr7.7757238.7759238 | 0.277 | 0.036 |
| LARC | NM_152997 | chr4.71090787.71092787 | 0.283 | 0.036 |
| LARC | NM_001166534 | chr5.55061712.55063712 | 0.307 | 0.036 |
| LARC | NM_001198783 | chr1.167297280.167299280 | 0.316 | 0.036 |
| LARC | NM_001277068 | chr2.228192393.228194393 | 0.32 | 0.036 |
| LARC | NM_006446 | chr12.21283127.21285127 | 0.327 | 0.036 |
| LARC | NM_001340 | chr9.105756592.105758592 | 0.329 | 0.036 |
| LARC | NM_014331 | chr4.139162503.139164503 | 0.339 | 0.036 |
| LARC | NM_001278300 | chr3.97157436.97159436 | 0.346 | 0.036 |
| LARC | NM_005295 | chr7.107109501.107111501 | 0.35 | 0.036 |
| LARC | NM_032997 | chr10.58120034.58122034 | 0.364 | 0.036 |
| LARC | NM_019098 | chr8.87754903.87756903 | 0.365 | 0.036 |
| LARC | NM_001005491 | chr11.55734939.55736939 | 0.366 | 0.036 |
| LARC | NM_001172173 | chr2.166325156.166327156 | 0.368 | 0.036 |
| LARC | NM_001323562 | chr20.5985583.5987583 | 0.374 | 0.036 |
| LARC | NM_001563 | chr6.76781395.76783395 | 0.376 | 0.036 |
| LARC | NM_001286771 | chr4.74087831.74089831 | 0.38 | 0.036 |
| LARC | NM_001128923 | chr4.152146660.152148660 | 0.382 | 0.036 |
| LARC | NM_182592 | chr4.44652658.44654658 | 0.386 | 0.036 |
| LARC | NM_001161834 | chr7.50134681.50136681 | 0.386 | 0.036 |
| LARC | NM_001010893 | chr8.82606207.82608207 | 0.39 | 0.036 |
| LARC | NM_016571 | chr6.64028882.64030882 | 0.393 | 0.036 |
| LARC | NM_001243349 | chr4.152148182.152150182 | 0.393 | 0.036 |
| LARC | NM_020194 | chr2.228191227.228193227 | 0.395 | 0.036 |
| LARC | NM_019109 | chr16.5120809.5122809 | 0.402 | 0.036 |
| LARC | NM_005409 | chr4.76956350.76958350 | 0.403 | 0.036 |
| LARC | NM_133339 | chr5.68666280.68668280 | 0.405 | 0.036 |
| LARC | NM_001297704 | chr1.82164454.82166454 | 0.408 | 0.036 |
| LARC | NM_080387 | chr12.8665135.8667135 | 0.411 | 0.036 |
| LARC | NM_001261830 | chr18.61444010.61446010 | 0.412 | 0.036 |
| LARC | NM_172058 | chr8.72267924.72269924 | 0.415 | 0.036 |
| LARC | NM_001242668 | chr8.94178079.94180079 | 0.42 | 0.036 |
| LARC | NM_182830 | chr14.47811449.47813449 | 0.424 | 0.036 |
| LARC | NM_002016 | chr1.152296679.152298679 | 0.425 | 0.036 |
| LARC | NM_001166696 | chr5.36605456.36607456 | 0.426 | 0.036 |
| LARC | NM_001128427 | chr4.163084186.163086186 | 0.427 | 0.036 |
| LARC | NM_001184696 | chr4.88753120.88755120 | 0.43 | 0.036 |
| LARC | NM_004811 | chr11.58342390.58344390 | 0.432 | 0.036 |
| LARC | NM_001162435 | chr6.46713653.46715653 | 0.432 | 0.036 |
| LARC | NM_023918 | chr12.10958579.10960579 | 0.433 | 0.036 |
| LARC | NM_001131005 | chr5.88198922.88200922 | 0.435 | 0.036 |
| LARC | NM_016331 | chr3.179040550.179042550 | 0.435 | 0.036 |
| LARC | NM_001352446 | chr10.35483777.35485777 | 0.436 | 0.036 |
| LARC | NM_173353 | chr12.72331625.72333625 | 0.437 | 0.036 |
| LARC | NM_173629 | chr18.52257389.52259389 | 0.438 | 0.036 |
| LARC | NM_001142541 | chr6.8434800.8436800 | 0.44 | 0.036 |
| LARC | NM_001004701 | chr11.55338603.55340603 | 0.441 | 0.036 |
| LARC | NM_012431 | chr7.83277479.83279479 | 0.446 | 0.036 |
| LARC | NM_001004747 | chr11.56018675.56020675 | 0.446 | 0.036 |
| LARC | NM_001198628 | chr8.93110916.93112916 | 0.447 | 0.036 |
| LARC | NM_001286754 | chr4.119770842.119772842 | 0.449 | 0.036 |
| LARC | NM_001243232 | chr18.53070226.53072226 | 0.451 | 0.036 |
| LARC | NM_001099746 | chr8.110692234.110694234 | 0.453 | 0.036 |
| LARC | NM_207365 | chr3.151450703.151452703 | 0.454 | 0.036 |
| LARC | NM_176892 | chr3.121795695.121797695 | 0.457 | 0.036 |
| LARC | NM_001122742 | chr6.152010630.152012630 | 0.457 | 0.036 |
| LARC | NM_017707 | chr1.23809750.23811750 | 0.461 | 0.036 |
| LARC | NM_001172626 | chr1.115237239.115239239 | 0.463 | 0.036 |
| LARC | NM_001164397 | chr11.89608185.89610185 | 0.469 | 0.036 |
| LARC | NM_032773 | chr3.197517096.197519096 | 0.47 | 0.036 |
| LARC | NM_153696 | chr11.89391464.89393464 | 0.472 | 0.036 |
| LARC | NM_001328609 | chr1.117296051.117298051 | 0.472 | 0.036 |
| LARC | NM_001329426 | chr7.93534819.93536819 | 0.474 | 0.036 |
| LARC | NM_001321548 | chr2.201373817.201375817 | 0.477 | 0.036 |
| LARC | NM_001004451 | chr9.125239205.125241205 | 0.482 | 0.036 |
| LARC | NM_001304526 | chr2.88354320.88356320 | 0.482 | 0.036 |
| LARC | NM_018130 | chr3.72896598.72898598 | 0.489 | 0.036 |
| LARC | NM_024727 | chr3.169586723.169588723 | 0.49 | 0.036 |
| LARC | NM_001001956 | chr9.107379485.107381485 | 0.49 | 0.036 |
| LARC | NM_001012729 | chr19.57677856.57679856 | 0.493 | 0.036 |
| LARC | NM_000193 | chr7.155603967.155605967 | 0.493 | 0.036 |
| LARC | NM_001270497 | chr20.45141211.45143211 | 0.495 | 0.036 |
| LARC | NM_001351193 | chr10.104912578.104914578 | 0.496 | 0.036 |
| LARC | NM_023921 | chr12.10977868.10979868 | 0.497 | 0.036 |
| LARC | NM_001349736 | chr5.149471187.149473187 | 0.497 | 0.036 |
| LARC | NM_001282556 | chr3.108014336.108016336 | 0.498 | 0.036 |
| LARC | NM_001002915 | chr19.46650038.46652038 | 0.5 | 0.036 |
| LARC | NM_030973 | chr19.50320535.50322535 | 0.501 | 0.036 |
| LARC | NM_139175 | chr7.122338208.122340208 | 0.502 | 0.036 |
| LARC | NM_033032 | chr17.39210463.39212463 | 0.505 | 0.036 |
| LARC | NM_001172640 | chr11.47509576.47511576 | 0.506 | 0.036 |
| LARC | NM_001277991 | chr1.115575566.115577566 | 0.507 | 0.036 |
| LARC | NM_207186 | chr11.6896855.6898855 | 0.514 | 0.036 |
| LARC | NM_005330 | chr11.5290373.5292373 | 0.515 | 0.036 |
| LARC | NM_001281972 | chr19.55343130.55345130 | 0.518 | 0.036 |
| LARC | NM_001243609 | chr12.16760148.16762148 | 0.518 | 0.036 |
| LARC | NM_052882 | chr19.57655570.57657570 | 0.52 | 0.036 |
| LARC | NM_175929 | chr13.103053124.103055124 | 0.522 | 0.036 |
| LARC | NM_001303615 | chr11.19080564.19082564 | 0.524 | 0.036 |
| LARC | NM_174908 | chr3.191045873.191047873 | 0.524 | 0.036 |
| LARC | NM_001127462 | chr1.247580350.247582350 | 0.524 | 0.036 |
| LARC | NM_001348952 | chr3.167370771.167372771 | 0.524 | 0.036 |
| LARC | NM_181700 | chr11.111636169.111638169 | 0.529 | 0.036 |
| LARC | NM_005244 | chr20.45522262.45524262 | 0.531 | 0.036 |
| LARC | NM_207398 | chr1.89640723.89642723 | 0.534 | 0.036 |
| LARC | NM_001135113 | chr19.46650499.46652499 | 0.54 | 0.036 |
| LARC | NM_198185 | chr11.7726941.7728941 | 0.543 | 0.036 |
| LARC | NM_018189 | chr3.109055419.109057419 | 0.546 | 0.036 |
| LARC | NM_001257317 | chr8.105351023.105353023 | 0.546 | 0.036 |
| LARC | NM_001348294 | chr6.29067619.29069619 | 0.549 | 0.036 |
| LARC | NM_001308088 | chr5.119866158.119868158 | 0.563 | 0.036 |
| LARC | NM_001099756 | chr8.110654419.110656419 | 0.564 | 0.036 |
| LARC | NM_030901 | chr19.14991167.14993167 | 0.567 | 0.036 |
| LARC | NM_005025 | chr3.167452510.167454510 | 0.568 | 0.036 |
| LARC | NM_001122752 | chr3.167452431.167454431 | 0.576 | 0.036 |
| LARC | NM_001270699 | chr10.32196854.32198854 | 0.582 | 0.036 |
| LARC | NM_001267610 | chr1.168105905.168107905 | 0.584 | 0.036 |
| LARC | NM_001318714 | chr16.57815281.57817281 | 0.585 | 0.036 |
| LARC | NM_001145110 | chr12.45306711.45308711 | 0.588 | 0.036 |
| LARC | NM_058207 | chr8.7308887.7310887 | 0.598 | 0.036 |
| LARC | NM_001195279 | chr3.12585963.12587963 | 0.6 | 0.036 |
| LARC | NM_017653 | chr18.46986103.46988103 | 0.601 | 0.036 |
| LARC | NM_198186 | chr9.119448494.119450494 | 0.61 | 0.036 |
| LARC | NM_153364 | chr12.100040527.100042527 | 0.614 | 0.036 |
| LARC | NM_001292035 | chr6.149538059.149540059 | 0.658 | 0.036 |
| LARC | NM_001013661 | chr1.159831447.159833447 | 1.52 | 0.036 |
| LARC | NM_006533 | chr19.41280281.41282281 | 1.589 | 0.036 |
| LARC | NM_001277378 | chr19.14195607.14197607 | 1.607 | 0.036 |
| LARC | NM_001310159 | chr11.31827473.31829473 | 1.619 | 0.036 |
| LARC | NM_014435 | chr4.76861166.76863166 | 1.63 | 0.036 |
| LARC | NM_000290 | chr7.44104186.44106186 | 1.674 | 0.036 |
| LARC | NM_001322114 | chr4.115519440.115521440 | 1.678 | 0.036 |
| LARC | NM_001278074 | chr9.137532650.137534650 | 1.692 | 0.036 |
| LARC | NM_001146254 | chr19.14063204.14065204 | 1.741 | 0.036 |
| LARC | NM_001002036 | chr2.96803175.96805175 | 1.746 | 0.036 |
| LARC | NM_001543 | chr5.149886673.149888673 | 1.757 | 0.036 |
| LARC | NM_057157 | chr10.94832231.94834231 | 1.758 | 0.036 |
| LARC | NM_016445 | chr14.67877917.67879917 | 1.761 | 0.036 |
| LARC | NM_001030060 | chr6.147828827.147830827 | 1.794 | 0.036 |
| LARC | NM_001258 | chr17.73995986.73997986 | 1.799 | 0.036 |
| LARC | NM_006288 | chr11.119293838.119295838 | 1.828 | 0.036 |
| LARC | NM_001348781 | chr17.14139179.14141179 | 1.829 | 0.036 |
| LARC | NM_020431 | chr14.77647101.77649101 | 1.838 | 0.036 |
| LARC | NM_024821 | chr22.42195625.42197625 | 1.847 | 0.036 |
| LARC | NM_001286826 | chr19.18117976.18119976 | 1.855 | 0.036 |
| LARC | NM_178469 | chr9.139651731.139653731 | 1.874 | 0.036 |
| LARC | NM_025196 | chr4.7068937.7070937 | 1.893 | 0.036 |
| LARC | NM_013358 | chr1.17530620.17532620 | 1.909 | 0.036 |
| LARC | NM_057176 | chr1.55463616.55465616 | 1.914 | 0.036 |
| LARC | NM_001354602 | chr1.43249702.43251702 | 1.914 | 0.036 |
| LARC | NM_001002879 | chr22.29948644.29950644 | 1.926 | 0.036 |
| LARC | NM_020777 | chr4.7193373.7195373 | 1.941 | 0.036 |
| LARC | NM_001207037 | chr6.36354577.36356577 | 1.951 | 0.036 |
| LARC | NM_001142674 | chr11.909874.911874 | 1.963 | 0.036 |
| LARC | NM_001166107 | chr1.120310555.120312555 | 1.972 | 0.036 |
| LARC | NM_001302510 | chr16.55512909.55514909 | 2.017 | 0.036 |
| LARC | NM_001199672 | chr7.128378345.128380345 | 2.022 | 0.036 |
| LARC | NM_001350402 | chr1.33546778.33548778 | 2.031 | 0.036 |
| LARC | NM_003678 | chr22.29948736.29950736 | 2.042 | 0.036 |
| LARC | NM_001318366 | chr10.99204774.99206774 | 2.052 | 0.036 |
| LARC | NM_002528 | chr16.2096870.2098870 | 2.072 | 0.036 |
| LARC | NM_001178009 | chr21.44495502.44497502 | 2.12 | 0.036 |
| LARC | NM_001329607 | chr15.64647458.64649458 | 2.138 | 0.036 |
| LARC | NM_001319952 | chr1.110305644.110307644 | 2.142 | 0.036 |
| LARC | NM_031459 | chr1.28584962.28586962 | 2.144 | 0.036 |
| LARC | NM_001008409 | chr20.30457439.30459439 | 2.223 | 0.036 |
| LARC | NM_198043 | chr10.99204887.99206887 | 2.23 | 0.036 |
| LARC | NM_014368 | chr9.124990091.124992091 | 2.242 | 0.036 |
| LARC | NM_001278635 | chr5.1886098.1888098 | 2.255 | 0.036 |
| LARC | NM_001304791 | chr1.11713431.11715431 | 2.3 | 0.036 |
| LARC | NM_001195141 | chr5.149736201.149738201 | 2.31 | 0.036 |
| LARC | NM_018346 | chr17.48555160.48557160 | 2.327 | 0.036 |
| LARC | NM_001144996 | chr12.56100686.56102686 | 2.348 | 0.036 |
| LARC | NM_001351027 | chr7.149743804.149745804 | 2.359 | 0.036 |
| LARC | NM_002254 | chr2.26204443.26206443 | 2.368 | 0.036 |
| LARC | NM_030629 | chr16.81527953.81529953 | 2.391 | 0.036 |
| LARC | NM_177478 | chr5.121186649.121188649 | 2.427 | 0.036 |
| LARC | NM_006869 | chr7.993319.995319 | 2.532 | 0.036 |
| LARC | NM_001160372 | chr8.141466861.141468861 | 2.557 | 0.036 |
| LARC | NM_017551 | chr10.88125250.88127250 | 2.561 | 0.036 |
| LARC | NM_144976 | chr19.12661356.12663356 | 2.578 | 0.036 |
| LARC | NM_001323312 | chr8.30889710.30891710 | 2.608 | 0.036 |
| LARC | NM_033437 | chr4.120548239.120550239 | 2.657 | 0.036 |
| LARC | NM_001321909 | chr2.115918683.115920683 | 2.663 | 0.036 |
| LARC | NM_001320352 | chr21.45844411.45846411 | 2.716 | 0.036 |
| LARC | NM_183372 | chr1.147623469.147625469 | 2.91 | 0.036 |
| LARC | NM_001134647 | chr4.7940653.7942653 | 3.017 | 0.036 |
| LARC | NM_014869 | chr3.13008198.13010198 | 3.03 | 0.036 |
| LARC | NM_138392 | chr19.41081756.41083756 | 3.407 | 0.036 |
| LARC | NM_001126102 | chr16.72087490.72089490 | 0.318 | 0.036 |
| LARC | NM_001014975 | chr1.196620007.196622007 | 0.332 | 0.036 |
| LARC | NM_214711 | chr4.71018903.71020903 | 0.345 | 0.036 |
| LARC | NM_002772 | chr21.19774970.19776970 | 0.42 | 0.036 |
| LARC | NM_001621 | chr7.17337275.17339275 | 0.427 | 0.036 |
| LARC | NM_015224 | chr3.56697074.56699074 | 0.454 | 0.036 |
| LARC | NM_005533 | chr17.41157741.41159741 | 2.11 | 0.036 |
| LARC | NM_022356 | chr1.43231755.43233755 | 2.387 | 0.036 |
| LARC | NM_000987 | chr17.8285568.8287568 | 2.428 | 0.036 |
| LARC | NM_015175 | chr3.47020172.47022172 | 2.472 | 0.036 |
| LARC | NM_001242333 | chr9.124989707.124991707 | 2.544 | 0.036 |
| LARC | NM_003461 | chr7.143077359.143079359 | 2.846 | 0.036 |
| LARC | NM_020645 | chr11.9024596.9026596 | 2.886 | 0.036 |
| LARC | NM_032902 | chr8.145702364.145704364 | 3.138 | 0.036 |
| LARC | NM_001278593 | chr14.74550196.74552196 | 0.313 | 0.037 |
| LARC | NM_001317918 | chr8.124779678.124781678 | 0.394 | 0.037 |
| LARC | NM_173591 | chr12.80602232.80604232 | 0.401 | 0.037 |
| LARC | NM_005230 | chr12.96587159.96589159 | 0.425 | 0.037 |
| LARC | NM_175060 | chr14.38724575.38726575 | 0.426 | 0.037 |
| LARC | NM_001937 | chr1.168697442.168699442 | 0.429 | 0.037 |
| LARC | NM_001101338 | chr9.115773472.115775472 | 0.44 | 0.037 |
| LARC | NM_033663 | chr3.113896899.113898899 | 0.451 | 0.037 |
| LARC | NM_016816 | chr12.113343581.113345581 | 0.451 | 0.037 |
| LARC | NM_001163558 | chr6.22302082.22304082 | 0.453 | 0.037 |
| LARC | NM_001144919 | chr10.123356972.123358972 | 0.473 | 0.037 |
| LARC | NM_001201380 | chr9.43683884.43685884 | 0.492 | 0.037 |
| LARC | NM_001005515 | chr3.97886543.97888543 | 0.521 | 0.037 |
| LARC | NM_001314051 | chr8.108347743.108349743 | 0.522 | 0.037 |
| LARC | NM_001001960 | chr11.55681058.55683058 | 0.532 | 0.037 |
| LARC | NM_006028 | chr11.113774517.113776517 | 0.533 | 0.037 |
| LARC | NM_021191 | chr12.55412728.55414728 | 0.537 | 0.037 |
| LARC | NM_173076 | chr2.216002151.216004151 | 0.54 | 0.037 |
| LARC | NM_001010939 | chr10.90345518.90347518 | 0.542 | 0.037 |
| LARC | NM_198584 | chr8.86156715.86158715 | 0.551 | 0.037 |
| LARC | NM_000198 | chr1.119956742.119958742 | 0.559 | 0.037 |
| LARC | NM_001242508 | chr18.21890470.21892470 | 0.567 | 0.037 |
| LARC | NM_001198943 | chr18.32396925.32398925 | 0.568 | 0.037 |
| LARC | NM_181604 | chr21.31970193.31972193 | 0.577 | 0.037 |
| LARC | NM_001099677 | chr8.12868772.12870772 | 0.578 | 0.037 |
| LARC | NM_001003799 | chr7.38312248.38314248 | 0.582 | 0.037 |
| LARC | NM_001351857 | chr11.7596997.7598997 | 0.601 | 0.037 |
| LARC | NM_001130960 | chr3.155393105.155395105 | 0.622 | 0.037 |
| LARC | NM_181876 | chr4.6382597.6384597 | 1.541 | 0.037 |
| LARC | NM_001039706 | chr7.89873487.89875487 | 1.581 | 0.037 |
| LARC | NM_002701 | chr6.31137470.31139470 | 1.611 | 0.037 |
| LARC | NM_212460 | chr7.12725451.12727451 | 1.679 | 0.037 |
| LARC | NM_001163121 | chr17.34256780.34258780 | 1.686 | 0.037 |
| LARC | NM_012326 | chr2.27192238.27194238 | 1.757 | 0.037 |
| LARC | NM_024092 | chr11.60680370.60682370 | 1.815 | 0.037 |
| LARC | NM_001142281 | chr11.118442101.118444101 | 1.854 | 0.037 |
| LARC | NM_147163 | chr18.12657737.12659737 | 1.9 | 0.037 |
| LARC | NM_001145641 | chr19.44115252.44117252 | 1.934 | 0.037 |
| LARC | NM_145912 | chr22.42827401.42829401 | 1.934 | 0.037 |
| LARC | NM_001321073 | chr21.44495541.44497541 | 1.953 | 0.037 |
| LARC | NM_130781 | chr5.176729745.176731745 | 2.035 | 0.037 |
| LARC | NM_001168390 | chr10.73496581.73498581 | 2.059 | 0.037 |
| LARC | NM_145726 | chr14.103242815.103244815 | 2.182 | 0.037 |
| LARC | NM_007068 | chr22.38965201.38967201 | 2.214 | 0.037 |
| LARC | NM_001349128 | chr1.44456518.44458518 | 2.255 | 0.037 |
| LARC | NM_001171511 | chr2.47167994.47169994 | 2.302 | 0.037 |
| LARC | NM_001282405 | chr10.64892006.64894006 | 2.319 | 0.037 |
| LARC | NM_003370 | chr19.46009687.46011687 | 2.319 | 0.037 |
| LARC | NM_001317203 | chr5.139027509.139029509 | 2.363 | 0.037 |
| LARC | NM_033225 | chr8.4851328.4853328 | 2.388 | 0.037 |
| LARC | NM_001146008 | chr8.70744625.70746625 | 2.447 | 0.037 |
| LARC | NM_001515 | chr5.70362497.70364497 | 2.518 | 0.037 |
| LARC | NM_001297437 | chr5.41924353.41926353 | 2.601 | 0.037 |
| LARC | NM_012176 | chr5.41924355.41926355 | 2.601 | 0.037 |
| LARC | NM_001287144 | chr8.81082894.81084894 | 2.627 | 0.037 |
| LARC | NM_000365 | chr12.6975692.6977692 | 2.641 | 0.037 |
| LARC | NM_001287008 | chr9.95819969.95821969 | 2.731 | 0.037 |
| LARC | NM_170784 | chr20.10413887.10415887 | 2.742 | 0.037 |
| LARC | NM_213594 | chr12.106975684.106977684 | 3.267 | 0.037 |
| LARC | NM_017801 | chr3.32543403.32545403 | 4.234 | 0.037 |
| LARC | NM_001098200 | chr13.99909682.99911682 | 0.354 | 0.038 |
| LARC | NM_001286731 | chr4.71383288.71385288 | 0.386 | 0.038 |
| LARC | NM_006899 | chr20.2643869.2645869 | 0.388 | 0.038 |
| LARC | NM_001442 | chr8.82394473.82396473 | 0.394 | 0.038 |
| LARC | NM_080284 | chr17.67137015.67139015 | 0.397 | 0.038 |
| LARC | NM_006583 | chr4.110748149.110750149 | 0.416 | 0.038 |
| LARC | NM_001349465 | chr15.25199138.25201138 | 0.418 | 0.038 |
| LARC | NM_001526 | chr6.54970257.54972257 | 0.419 | 0.038 |
| LARC | NM_001193421 | chr20.51800821.51802821 | 0.428 | 0.038 |
| LARC | NM_001145838 | chr8.66581107.66583107 | 0.445 | 0.038 |
| LARC | NM_001251829 | chr4.88895801.88897801 | 0.458 | 0.038 |
| LARC | NM_001348344 | chr3.120626578.120628578 | 0.461 | 0.038 |
| LARC | NM_052913 | chr6.130757261.130759261 | 0.463 | 0.038 |
| LARC | NM_020161 | chr2.228497036.228499036 | 0.484 | 0.038 |
| LARC | NM_002260 | chr12.10587592.10589592 | 0.486 | 0.038 |
| LARC | NM_003814 | chr14.71000732.71002732 | 0.49 | 0.038 |
| LARC | NM_007176 | chr14.76126538.76128538 | 0.49 | 0.038 |
| LARC | NM_001290232 | chr15.36886069.36888069 | 0.501 | 0.038 |
| LARC | NM_052947 | chr18.56295189.56297189 | 0.503 | 0.038 |
| LARC | NM_001297650 | chr12.7595781.7597781 | 0.507 | 0.038 |
| LARC | NM_145175 | chr2.14771806.14773806 | 0.508 | 0.038 |
| LARC | NM_020770 | chr1.151482861.151484861 | 0.51 | 0.038 |
| LARC | NM_007126 | chr9.35071739.35073739 | 0.51 | 0.038 |
| LARC | NM_001100396 | chr2.54557070.54559070 | 0.512 | 0.038 |
| LARC | NM_018405 | chr17.30185326.30187326 | 0.514 | 0.038 |
| LARC | NM_001282583 | chr1.1296157.1298157 | 0.516 | 0.038 |
| LARC | NM_001321949 | chr13.103053778.103055778 | 0.518 | 0.038 |
| LARC | NM_138327 | chr6.132966165.132968165 | 0.519 | 0.038 |
| LARC | NM_001355223 | chr1.248603417.248605417 | 0.52 | 0.038 |
| LARC | NM_001166120 | chr1.119956553.119958553 | 0.52 | 0.038 |
| LARC | NM_001316903 | chr19.36194388.36196388 | 0.52 | 0.038 |
| LARC | NM_001290809 | chr3.113896623.113898623 | 0.526 | 0.038 |
| LARC | NM_001166687 | chr12.48515429.48517429 | 0.533 | 0.038 |
| LARC | NM_001303228 | chr4.39033041.39035041 | 0.54 | 0.038 |
| LARC | NM_033187 | chr17.39323424.39325424 | 0.546 | 0.038 |
| LARC | NM_001278433 | chr17.66408763.66410763 | 0.558 | 0.038 |
| LARC | NM_170706 | chr1.183273009.183275009 | 0.561 | 0.038 |
| LARC | NM_001111031 | chr2.158453072.158455072 | 0.576 | 0.038 |
| LARC | NM_002022 | chr1.171282321.171284321 | 0.581 | 0.038 |
| LARC | NM_001005566 | chr11.58189786.58191786 | 0.59 | 0.038 |
| LARC | NM_001002925 | chr11.56408915.56410915 | 0.59 | 0.038 |
| LARC | NM_001005567 | chr11.5525882.5527882 | 0.605 | 0.038 |
| LARC | NM_001004319 | chr1.156268428.156270428 | 0.612 | 0.038 |
| LARC | NM_001244960 | chr9.86081657.86083657 | 0.618 | 0.038 |
| LARC | NM_005752 | chr16.78055442.78057442 | 0.621 | 0.038 |
| LARC | NM_001184734 | chr9.119448542.119450542 | 0.642 | 0.038 |
| LARC | NM_001242329 | chr4.9363854.9365854 | 1.548 | 0.038 |
| LARC | NM_001322305 | chr1.224803178.224805178 | 1.672 | 0.038 |
| LARC | NM_178491 | chr20.42964797.42966797 | 1.687 | 0.038 |
| LARC | NM_001162427 | chr9.135819020.135821020 | 1.691 | 0.038 |
| LARC | NM_001007089 | chr2.220196899.220198899 | 1.699 | 0.038 |
| LARC | NM_001184780 | chr15.69221838.69223838 | 1.716 | 0.038 |
| LARC | NM_013447 | chr19.14888353.14890353 | 1.802 | 0.038 |
| LARC | NM_006841 | chr3.50241678.50243678 | 1.833 | 0.038 |
| LARC | NM_001305116 | chr8.33454315.33456315 | 1.858 | 0.038 |
| LARC | NM_033414 | chr5.16464894.16466894 | 1.878 | 0.038 |
| LARC | NM_014978 | chr10.106399858.106401858 | 1.888 | 0.038 |
| LARC | NM_001350237 | chr1.109824766.109826766 | 1.9 | 0.038 |
| LARC | NM_032636 | chr1.109824790.109826790 | 1.9 | 0.038 |
| LARC | NM_001037162 | chr14.74082547.74084547 | 1.917 | 0.038 |
| LARC | NM_002224 | chr6.33588155.33590155 | 1.952 | 0.038 |
| LARC | NM_001163990 | chr17.72732355.72734355 | 1.953 | 0.038 |
| LARC | NM_005356 | chr1.32715839.32717839 | 2.052 | 0.038 |
| LARC | NM_001330524 | chr19.43382974.43384974 | 2.081 | 0.038 |
| LARC | NM_013446 | chr7.140178369.140180369 | 2.087 | 0.038 |
| LARC | NM_003770 | chr17.39579822.39581822 | 2.106 | 0.038 |
| LARC | NM_000348 | chr2.31805040.31807040 | 2.172 | 0.038 |
| LARC | NM_001270893 | chr19.45680501.45682501 | 2.244 | 0.038 |
| LARC | NM_001303254 | chr15.74043711.74045711 | 2.316 | 0.038 |
| LARC | NM_004158 | chr19.6374927.6376927 | 2.328 | 0.038 |
| LARC | NM_001197123 | chr19.50168132.50170132 | 2.332 | 0.038 |
| LARC | NM_002888 | chr3.158449275.158451275 | 2.417 | 0.038 |
| LARC | NM_015512 | chr3.52349334.52351334 | 2.458 | 0.038 |
| LARC | NM_053056 | chr11.69454872.69456872 | 2.482 | 0.038 |
| LARC | NM_001281501 | chr11.61582674.61584674 | 2.6 | 0.038 |
| LARC | NM_130849 | chr8.145641279.145643279 | 2.744 | 0.038 |
| LARC | NM_014902 | chr20.34893244.34895244 | 2.946 | 0.038 |
| LARC | NM_006627 | chr19.30096169.30098169 | 0.345 | 0.039 |
| LARC | NM_001349372 | chr5.81573468.81575468 | 0.361 | 0.039 |
| LARC | NM_138716 | chr8.16049300.16051300 | 0.385 | 0.039 |
| LARC | NM_001354145 | chr14.39733475.39735475 | 0.391 | 0.039 |
| LARC | NM_001490 | chr9.79073067.79075067 | 0.428 | 0.039 |
| LARC | NM_016300 | chr3.35682848.35684848 | 0.43 | 0.039 |
| LARC | NM_032593 | chr9.35814042.35816042 | 0.441 | 0.039 |
| LARC | NM_001330504 | chr16.5121248.5123248 | 0.441 | 0.039 |
| LARC | NM_005588 | chr6.46760093.46762093 | 0.453 | 0.039 |
| LARC | NM_182720 | chr10.35483054.35485054 | 0.454 | 0.039 |
| LARC | NM_001297742 | chr11.13460826.13462826 | 0.458 | 0.039 |
| LARC | NM_001288986 | chr17.66950533.66952533 | 0.459 | 0.039 |
| LARC | NM_145268 | chr7.129846703.129848703 | 0.476 | 0.039 |
| LARC | NM_012108 | chr4.68423414.68425414 | 0.477 | 0.039 |
| LARC | NM_001320768 | chr21.27944723.27946723 | 0.489 | 0.039 |
| LARC | NM_001300930 | chr11.89866817.89868817 | 0.49 | 0.039 |
| LARC | NM_001206729 | chr1.244005886.244007886 | 0.49 | 0.039 |
| LARC | NM_206808 | chr13.100257917.100259917 | 0.49 | 0.039 |
| LARC | NM_032600 | chr3.107095187.107097187 | 0.491 | 0.039 |
| LARC | NM_032587 | chr5.40840409.40842409 | 0.494 | 0.039 |
| LARC | NM_001080491 | chr10.11573279.11575279 | 0.494 | 0.039 |
| LARC | NM_001291304 | chr7.123240705.123242705 | 0.503 | 0.039 |
| LARC | NM_001304384 | chr3.27257014.27259014 | 0.504 | 0.039 |
| LARC | NM_001122853 | chr5.150039402.150041402 | 0.508 | 0.039 |
| LARC | NM_013431 | chr12.10561356.10563356 | 0.51 | 0.039 |
| LARC | NM_001270934 | chr5.147161411.147163411 | 0.513 | 0.039 |
| LARC | NM_001146162 | chr11.89442466.89444466 | 0.515 | 0.039 |
| LARC | NM_001203245 | chr6.34392818.34394818 | 0.518 | 0.039 |
| LARC | NM_016037 | chr1.38477383.38479383 | 0.52 | 0.039 |
| LARC | NM_001005519 | chr12.55885161.55887161 | 0.52 | 0.039 |
| LARC | NM_001304532 | chr8.27629170.27631170 | 0.526 | 0.039 |
| LARC | NM_001290021 | chr10.28269846.28271846 | 0.538 | 0.039 |
| LARC | NM_002175 | chr9.21165659.21167659 | 0.539 | 0.039 |
| LARC | NM_080539 | chr3.15562258.15564258 | 0.541 | 0.039 |
| LARC | NM_004132 | chr10.115311736.115313736 | 0.545 | 0.039 |
| LARC | NM_001220484 | chr14.74024651.74026651 | 0.549 | 0.039 |
| LARC | NM_007008 | chr2.55236470.55238470 | 0.551 | 0.039 |
| LARC | NM_014106 | chr15.35279497.35281497 | 0.565 | 0.039 |
| LARC | NM_001286624 | chr21.30516879.30518879 | 0.57 | 0.039 |
| LARC | NM_001460 | chr1.171153346.171155346 | 0.573 | 0.039 |
| LARC | NM_001328646 | chr2.105945128.105947128 | 0.574 | 0.039 |
| LARC | NM_178455 | chr20.44349973.44351973 | 0.576 | 0.039 |
| LARC | NM_058244 | chr5.137418580.137420580 | 0.58 | 0.039 |
| LARC | NM_001349272 | chr3.129406575.129408575 | 0.583 | 0.039 |
| LARC | NM_024415 | chr5.55032844.55034844 | 0.592 | 0.039 |
| LARC | NM_000689 | chr9.75567233.75569233 | 0.595 | 0.039 |
| LARC | NM_171830 | chr3.178968645.178970645 | 0.605 | 0.039 |
| LARC | NM_004063 | chr8.95219815.95221815 | 0.612 | 0.039 |
| LARC | NM_001145350 | chr20.34116481.34118481 | 0.626 | 0.039 |
| LARC | NM_020641 | chr9.27296137.27298137 | 0.639 | 0.039 |
| LARC | NM_004590 | chr17.34307532.34309532 | 0.657 | 0.039 |
| LARC | NM_001356338 | chr21.41013311.41015311 | 0.661 | 0.039 |
| LARC | NM_012210 | chr9.119448580.119450580 | 0.663 | 0.039 |
| LARC | NM_001127702 | chr14.94856029.94858029 | 1.524 | 0.039 |
| LARC | NM_001199887 | chr8.79716758.79718758 | 1.526 | 0.039 |
| LARC | NM_001202553 | chr19.41280081.41282081 | 1.562 | 0.039 |
| LARC | NM_002463 | chr21.42732949.42734949 | 1.575 | 0.039 |
| LARC | NM_001347915 | chr14.54419193.54421193 | 1.623 | 0.039 |
| LARC | NM_001242908 | chr1.38099595.38101595 | 1.623 | 0.039 |
| LARC | NM_000326 | chr15.89763922.89765922 | 1.627 | 0.039 |
| LARC | NM_001004712 | chr14.20482352.20484352 | 1.645 | 0.039 |
| LARC | NM_001284509 | chr17.6615740.6617740 | 1.668 | 0.039 |
| LARC | NM_015264 | chr22.45607347.45609347 | 1.7 | 0.039 |
| LARC | NM_001242811 | chr6.90271036.90273036 | 1.724 | 0.039 |
| LARC | NM_005512 | chr11.76380044.76382044 | 1.725 | 0.039 |
| LARC | NM_001017404 | chr1.202182282.202184282 | 1.766 | 0.039 |
| LARC | NM_174938 | chr9.86152348.86154348 | 1.827 | 0.039 |
| LARC | NM_001301820 | chr16.31469174.31471174 | 1.834 | 0.039 |
| LARC | NM_017956 | chr8.125462047.125464047 | 1.894 | 0.039 |
| LARC | NM_001349921 | chr17.41149289.41151289 | 1.897 | 0.039 |
| LARC | NM_001351264 | chr17.71227228.71229228 | 1.903 | 0.039 |
| LARC | NM_001205252 | chr1.1008687.1010687 | 1.942 | 0.039 |
| LARC | NM_005564 | chr9.130910708.130912708 | 1.97 | 0.039 |
| LARC | NM_032961 | chr4.134069444.134071444 | 1.978 | 0.039 |
| LARC | NM_001195278 | chr7.140773031.140775031 | 1.99 | 0.039 |
| LARC | NM_001166262 | chr13.28023326.28025326 | 1.993 | 0.039 |
| LARC | NM_014940 | chr16.77223815.77225815 | 2.007 | 0.039 |
| LARC | NM_007260 | chr1.24116645.24118645 | 2.031 | 0.039 |
| LARC | NM_018214 | chr6.53658777.53660777 | 2.04 | 0.039 |
| LARC | NM_001278784 | chr17.47784313.47786313 | 2.073 | 0.039 |
| LARC | NM_032479 | chr5.1798956.1800956 | 2.085 | 0.039 |
| LARC | NM_000071 | chr21.44495040.44497040 | 2.091 | 0.039 |
| LARC | NM_001354009 | chr21.44495055.44497055 | 2.091 | 0.039 |
| LARC | NM_001349127 | chr1.44456267.44458267 | 2.101 | 0.039 |
| LARC | NM_173518 | chr8.67781983.67783983 | 2.112 | 0.039 |
| LARC | NM_013357 | chr8.30889317.30891317 | 2.139 | 0.039 |
| LARC | NM_014241 | chr10.17658373.17660373 | 2.153 | 0.039 |
| LARC | NM_016364 | chr10.76858248.76860248 | 2.179 | 0.039 |
| LARC | NM_001322832 | chr10.79396577.79398577 | 2.213 | 0.039 |
| LARC | NM_021127 | chr18.57566191.57568191 | 2.218 | 0.039 |
| LARC | NM_001134888 | chr14.101350184.101352184 | 2.236 | 0.039 |
| LARC | NM_024097 | chr1.43231915.43233915 | 2.265 | 0.039 |
| LARC | NM_001336 | chr20.57581309.57583309 | 2.308 | 0.039 |
| LARC | NM_015044 | chr16.23520815.23522815 | 2.327 | 0.039 |
| LARC | NM_198977 | chr19.42386266.42388266 | 2.347 | 0.039 |
| LARC | NM_145062 | chr6.116988973.116990973 | 2.365 | 0.039 |
| LARC | NM_001127649 | chr22.18559759.18561759 | 2.42 | 0.039 |
| LARC | NM_033339 | chr10.115438427.115440427 | 2.539 | 0.039 |
| LARC | NM_004207 | chr17.80185886.80187886 | 2.642 | 0.039 |
| LARC | NM_148415 | chr16.28833368.28835368 | 2.673 | 0.039 |
| LARC | NM_018143 | chr17.40020684.40022684 | 2.694 | 0.039 |
| LARC | NM_001270695 | chr10.32216804.32218804 | 2.851 | 0.039 |
| LARC | NM_031454 | chr22.50638407.50640407 | 2.923 | 0.039 |
| LARC | NM_001281502 | chr11.61582875.61584875 | 2.883 | 0.04 |
| LARC | NM_001324080 | chr1.222912025.222914025 | 0.383 | 0.041 |
| LARC | NM_002054 | chr2.163007914.163009914 | 0.411 | 0.041 |
| LARC | NM_001177306 | chr5.102200526.102202526 | 0.453 | 0.041 |
| LARC | NM_018315 | chr4.153273110.153275110 | 0.541 | 0.041 |
| LARC | NM_001142624 | chr17.27044286.27046286 | 1.752 | 0.041 |
| LARC | NM_001286818 | chr13.73628113.73630113 | 0.287 | 0.041 |
| LARC | NM_003242 | chr3.30646993.30648993 | 0.314 | 0.041 |
| LARC | NM_018989 | chr5.145582162.145584162 | 0.369 | 0.041 |
| LARC | NM_001037499 | chr6.49930818.49932818 | 0.381 | 0.041 |
| LARC | NM_001005205 | chr11.56126690.56128690 | 0.384 | 0.041 |
| LARC | NM_001042784 | chr4.77327458.77329458 | 0.387 | 0.041 |
| LARC | NM_144632 | chr2.103377489.103379489 | 0.394 | 0.041 |
| LARC | NM_025185 | chr17.61085897.61087897 | 0.413 | 0.041 |
| LARC | NM_025107 | chr6.153018029.153020029 | 0.418 | 0.041 |
| LARC | NM_000096 | chr3.148938832.148940832 | 0.422 | 0.041 |
| LARC | NM_000315 | chr11.13516567.13518567 | 0.424 | 0.041 |
| LARC | NM_001166504 | chr4.100355291.100357291 | 0.438 | 0.041 |
| LARC | NM_000586 | chr4.123376650.123378650 | 0.443 | 0.041 |
| LARC | NM_001244390 | chr9.34328503.34330503 | 0.445 | 0.041 |
| LARC | NM_001242676 | chr1.110545569.110547569 | 0.447 | 0.041 |
| LARC | NM_205841 | chr5.147581356.147583356 | 0.454 | 0.041 |
| LARC | NM_001285461 | chr5.75903919.75905919 | 0.469 | 0.041 |
| LARC | NM_001256267 | chr10.69864873.69866873 | 0.47 | 0.041 |
| LARC | NM_001025108 | chr2.100721045.100723045 | 0.472 | 0.041 |
| LARC | NM_173505 | chr18.21241849.21243849 | 0.477 | 0.041 |
| LARC | NM_001220479 | chr12.81762193.81764193 | 0.479 | 0.041 |
| LARC | NM_003679 | chr1.241694433.241696433 | 0.481 | 0.041 |
| LARC | NM_001040272 | chr9.18473078.18475078 | 0.484 | 0.041 |
| LARC | NM_130837 | chr3.193309932.193311932 | 0.499 | 0.041 |
| LARC | NM_014314 | chr9.32525322.32527322 | 0.505 | 0.041 |
| LARC | NM_197954 | chr12.10281868.10283868 | 0.506 | 0.041 |
| LARC | NM_194294 | chr8.39791473.39793473 | 0.513 | 0.041 |
| LARC | NM_001130928 | chr15.100172182.100174182 | 0.53 | 0.041 |
| LARC | NM_002820 | chr12.28123916.28125916 | 0.535 | 0.041 |
| LARC | NM_001170416 | chr9.71819077.71821077 | 0.537 | 0.041 |
| LARC | NM_015954 | chr12.16063105.16065105 | 0.542 | 0.041 |
| LARC | NM_001244583 | chr7.115607367.115609367 | 0.543 | 0.041 |
| LARC | NM_001278596 | chr12.6053425.6055425 | 0.544 | 0.041 |
| LARC | NM_001321940 | chr13.103053382.103055382 | 0.545 | 0.041 |
| LARC | NM_001321941 | chr13.103053384.103055384 | 0.545 | 0.041 |
| LARC | NM_001330615 | chr3.12352878.12354878 | 0.546 | 0.041 |
| LARC | NM_001136230 | chr4.88243058.88245058 | 0.552 | 0.041 |
| LARC | NM_004460 | chr2.163099067.163101067 | 0.555 | 0.041 |
| LARC | NM_203406 | chr5.89769585.89771585 | 0.556 | 0.041 |
| LARC | NM_001190945 | chr9.123690451.123692451 | 0.558 | 0.041 |
| LARC | NM_001143831 | chr11.88780240.88782240 | 0.56 | 0.041 |
| LARC | NM_017515 | chr11.107728914.107730914 | 0.562 | 0.041 |
| LARC | NM_001288634 | chr17.27138453.27140453 | 0.564 | 0.041 |
| LARC | NM_001320758 | chr2.231988824.231990824 | 0.571 | 0.041 |
| LARC | NM_001112704 | chr10.118896812.118898812 | 0.574 | 0.041 |
| LARC | NM_001348257 | chr11.5198941.5200941 | 0.575 | 0.041 |
| LARC | NM_001164279 | chr11.118900616.118902616 | 0.579 | 0.041 |
| LARC | NM_058222 | chr10.114042413.114044413 | 0.58 | 0.041 |
| LARC | NM_001267060 | chr2.27592362.27594362 | 0.586 | 0.041 |
| LARC | NM_001004723 | chr14.20270928.20272928 | 0.587 | 0.041 |
| LARC | NM_003149 | chr3.36420978.36422978 | 0.587 | 0.041 |
| LARC | NM_001164425 | chr19.7057645.7059645 | 0.59 | 0.041 |
| LARC | NM_003817 | chr8.24297508.24299508 | 0.591 | 0.041 |
| LARC | NM_001005479 | chr3.97982128.97984128 | 0.603 | 0.041 |
| LARC | NM_001266 | chr16.55866075.55868075 | 0.615 | 0.041 |
| LARC | NM_001321863 | chr2.55273022.55275022 | 0.623 | 0.041 |
| LARC | NM_001001923 | chr9.125550211.125552211 | 0.626 | 0.041 |
| LARC | NM_138694 | chr6.51951423.51953423 | 0.629 | 0.041 |
| LARC | NM_001302959 | chr11.67249986.67251986 | 0.644 | 0.041 |
| LARC | NM_001290226 | chr5.66253697.66255697 | 0.648 | 0.041 |
| LARC | NM_032730 | chr6.107076373.107078373 | 0.659 | 0.041 |
| LARC | NM_001723 | chr6.56506694.56508694 | 0.666 | 0.041 |
| LARC | NM_001242327 | chr4.9344873.9346873 | 1.514 | 0.041 |
| LARC | NM_006760 | chr11.118826007.118828007 | 1.659 | 0.041 |
| LARC | NM_198447 | chr1.204182220.204184220 | 1.688 | 0.041 |
| LARC | NM_001882 | chr5.76247679.76249679 | 1.7 | 0.041 |
| LARC | NM_145640 | chr22.36555977.36557977 | 1.707 | 0.041 |
| LARC | NM_001271707 | chr3.12597512.12599512 | 1.743 | 0.041 |
| LARC | NM_001173484 | chr10.21462116.21464116 | 1.749 | 0.041 |
| LARC | NM_021232 | chr19.36303201.36305201 | 1.772 | 0.041 |
| LARC | NM_001111034 | chr19.11688595.11690595 | 1.779 | 0.041 |
| LARC | NM_001315501 | chr11.2922649.2924649 | 1.842 | 0.041 |
| LARC | NM_001010 | chr9.19379235.19381235 | 1.863 | 0.041 |
| LARC | NM_173689 | chr9.126117445.126119445 | 1.868 | 0.041 |
| LARC | NM_000783 | chr10.94832646.94834646 | 1.871 | 0.041 |
| LARC | NM_052838 | chr16.30393171.30395171 | 1.879 | 0.041 |
| LARC | NM_001331024 | chr4.166127769.166129769 | 1.886 | 0.041 |
| LARC | NM_052916 | chr17.74235390.74237390 | 1.895 | 0.041 |
| LARC | NM_001330501 | chr17.74235392.74237392 | 1.895 | 0.041 |
| LARC | NM_017767 | chr8.145640917.145642917 | 1.897 | 0.041 |
| LARC | NM_001166261 | chr13.28023334.28025334 | 1.934 | 0.041 |
| LARC | NM_018719 | chr7.21984542.21986542 | 1.943 | 0.041 |
| LARC | NM_024681 | chr22.37446775.37448775 | 1.953 | 0.041 |
| LARC | NM_002555 | chr11.2922511.2924511 | 2.003 | 0.041 |
| LARC | NM_017908 | chr19.58986530.58988530 | 2.008 | 0.041 |
| LARC | NM_014077 | chr19.16295211.16297211 | 2.034 | 0.041 |
| LARC | NM_001353011 | chr15.77196798.77198798 | 2.05 | 0.041 |
| LARC | NM_025232 | chr8.21998464.22000464 | 2.088 | 0.041 |
| LARC | NM_005322 | chr6.27834359.27836359 | 2.098 | 0.041 |
| LARC | NM_003780 | chr1.44443873.44445873 | 2.143 | 0.041 |
| LARC | NM_001104595 | chr22.45703840.45705840 | 2.235 | 0.041 |
| LARC | NM_001135211 | chr7.72741154.72743154 | 2.263 | 0.041 |
| LARC | NM_001146009 | chr8.70744404.70746404 | 2.267 | 0.041 |
| LARC | NM_001270448 | chr17.7122149.7124149 | 2.329 | 0.041 |
| LARC | NM_001001557 | chr8.97172020.97174020 | 2.389 | 0.041 |
| LARC | NM_017929 | chr22.18559685.18561685 | 2.391 | 0.041 |
| LARC | NM_001136036 | chr1.249152125.249154125 | 2.401 | 0.041 |
| LARC | NM_001278431 | chr11.119210593.119212593 | 2.414 | 0.041 |
| LARC | NM_003586 | chr16.30021401.30023401 | 2.483 | 0.041 |
| LARC | NM_019020 | chr17.78008657.78010657 | 2.776 | 0.041 |
| LARC | NM_006180 | chr9.87282372.87284372 | 0.483 | 0.041 |
| LARC | NM_001114387 | chr4.68828232.68830232 | 0.549 | 0.041 |
| LARC | NM_015235 | chr10.53458355.53460355 | 0.632 | 0.041 |
| LARC | NM_004658 | chr12.113573044.113575044 | 1.534 | 0.041 |
| LARC | NM_001113239 | chr7.139476693.139478693 | 1.864 | 0.041 |
| LARC | NM_001300759 | chr5.114504660.114506660 | 2.301 | 0.041 |
| LARC | NM_014923 | chr13.49683438.49685438 | 0.308 | 0.042 |
| LARC | NM_006217 | chr3.167190920.167192920 | 0.362 | 0.042 |
| LARC | NM_144646 | chr4.71531348.71533348 | 0.366 | 0.042 |
| LARC | NM_001351287 | chr12.86650062.86652062 | 0.386 | 0.042 |
| LARC | NM_153184 | chr3.85774631.85776631 | 0.397 | 0.042 |
| LARC | NM_001146032 | chr5.72250807.72252807 | 0.434 | 0.042 |
| LARC | NM_138573 | chr15.76303785.76305785 | 0.435 | 0.042 |
| LARC | NM_003341 | chr3.23846383.23848383 | 0.443 | 0.042 |
| LARC | NM_001291379 | chr10.46348325.46350325 | 0.444 | 0.042 |
| LARC | NM_145865 | chr16.21244015.21246015 | 0.446 | 0.042 |
| LARC | NM_001303051 | chr1.202896772.202898772 | 0.448 | 0.042 |
| LARC | NM_004510 | chr2.231083827.231085827 | 0.449 | 0.042 |
| LARC | NM_001270410 | chr1.226496449.226498449 | 0.449 | 0.042 |
| LARC | NM_001004746 | chr11.55999661.56001661 | 0.469 | 0.042 |
| LARC | NM_001164435 | chr21.32090095.32092095 | 0.472 | 0.042 |
| LARC | NM_003097 | chr15.25199039.25201039 | 0.472 | 0.042 |
| LARC | NM_022804 | chr15.25199069.25201069 | 0.472 | 0.042 |
| LARC | NM_153694 | chr12.102132250.102134250 | 0.474 | 0.042 |
| LARC | NM_177542 | chr19.46194250.46196250 | 0.476 | 0.042 |
| LARC | NM_001195573 | chr14.95598840.95600840 | 0.481 | 0.042 |
| LARC | NM_182530 | chr12.70218102.70220102 | 0.483 | 0.042 |
| LARC | NM_001164830 | chr8.86252943.86254943 | 0.495 | 0.042 |
| LARC | NM_001145026 | chr12.80837125.80839125 | 0.496 | 0.042 |
| LARC | NM_001105580 | chr3.97753148.97755148 | 0.497 | 0.042 |
| LARC | NM_001314047 | chr9.6214148.6216148 | 0.501 | 0.042 |
| LARC | NM_181528 | chr20.19996933.19998933 | 0.501 | 0.042 |
| LARC | NM_001323564 | chr20.5986312.5988312 | 0.503 | 0.042 |
| LARC | NM_001146017 | chr6.43456321.43458321 | 0.503 | 0.042 |
| LARC | NM_001282390 | chr2.161055824.161057824 | 0.506 | 0.042 |
| LARC | NM_021268 | chr9.21227221.21229221 | 0.506 | 0.042 |
| LARC | NM_002170 | chr9.21408145.21410145 | 0.507 | 0.042 |
| LARC | NM_001354646 | chr12.14995467.14997467 | 0.507 | 0.042 |
| LARC | NM_024787 | chr8.33423646.33425646 | 0.512 | 0.042 |
| LARC | NM_001349455 | chr15.25067754.25069754 | 0.513 | 0.042 |
| LARC | NM_001004705 | chr11.59243902.59245902 | 0.513 | 0.042 |
| LARC | NM_001172105 | chr3.113615317.113617317 | 0.514 | 0.042 |
| LARC | NM_001345999 | chr5.37378361.37380361 | 0.514 | 0.042 |
| LARC | NM_052952 | chr2.189653831.189655831 | 0.52 | 0.042 |
| LARC | NM_001267706 | chr9.5449502.5451502 | 0.521 | 0.042 |
| LARC | NM_004163 | chr18.52494707.52496707 | 0.522 | 0.042 |
| LARC | NM_001145118 | chr7.6590067.6592067 | 0.524 | 0.042 |
| LARC | NM_018566 | chr1.207223422.207225422 | 0.525 | 0.042 |
| LARC | NM_001242925 | chr1.205197058.205199058 | 0.533 | 0.042 |
| LARC | NM_031962 | chr17.39387714.39389714 | 0.534 | 0.042 |
| LARC | NM_001004461 | chr11.7949209.7951209 | 0.538 | 0.042 |
| LARC | NM_001004488 | chr7.143770312.143772312 | 0.54 | 0.042 |
| LARC | NM_001278716 | chr6.99394882.99396882 | 0.543 | 0.042 |
| LARC | NM_000943 | chr5.122371425.122373425 | 0.544 | 0.042 |
| LARC | NM_198506 | chr4.110768339.110770339 | 0.544 | 0.042 |
| LARC | NM_022051 | chr1.231559790.231561790 | 0.55 | 0.042 |
| LARC | NM_000212 | chr17.45330207.45332207 | 0.55 | 0.042 |
| LARC | NM_178356 | chr1.152680522.152682522 | 0.551 | 0.042 |
| LARC | NM_001348208 | chr6.29383841.29385841 | 0.552 | 0.042 |
| LARC | NM_053283 | chr12.55041277.55043277 | 0.552 | 0.042 |
| LARC | NM_001004686 | chr1.248200473.248202473 | 0.555 | 0.042 |
| LARC | NM_015040 | chr2.209129990.209131990 | 0.56 | 0.042 |
| LARC | NM_031453 | chr10.14815896.14817896 | 0.563 | 0.042 |
| LARC | NM_152841 | chr22.26874647.26876647 | 0.564 | 0.042 |
| LARC | NM_176876 | chr3.151057643.151059643 | 0.565 | 0.042 |
| LARC | NM_001324187 | chr15.84158365.84160365 | 0.571 | 0.042 |
| LARC | NM_001184976 | chr12.54891567.54893567 | 0.574 | 0.042 |
| LARC | NM_001301139 | chr7.94284521.94286521 | 0.578 | 0.042 |
| LARC | NM_001025389 | chr11.10475665.10477665 | 0.578 | 0.042 |
| LARC | NM_001308176 | chr18.25615549.25617549 | 0.586 | 0.042 |
| LARC | NM_001129883 | chr11.125657005.125659005 | 0.586 | 0.042 |
| LARC | NM_001128227 | chr9.36276053.36278053 | 0.592 | 0.042 |
| LARC | NM_001271037 | chr1.161599837.161601837 | 0.598 | 0.042 |
| LARC | NM_000494 | chr10.105844638.105846638 | 0.606 | 0.042 |
| LARC | NM_032782 | chr5.156535248.156537248 | 0.607 | 0.042 |
| LARC | NM_005157 | chr9.133709641.133711641 | 0.611 | 0.042 |
| LARC | NM_138817 | chr8.87241604.87243604 | 0.614 | 0.042 |
| LARC | NM_182905 | chr9.28739.30739 | 0.624 | 0.042 |
| LARC | NM_005836 | chr8.99128418.99130418 | 0.626 | 0.042 |
| LARC | NM_207469 | chr20.237376.239376 | 0.629 | 0.042 |
| LARC | NM_001005163 | chr11.5508914.5510914 | 0.634 | 0.042 |
| LARC | NM_001267614 | chr1.168105811.168107811 | 0.644 | 0.042 |
| LARC | NM_001289947 | chr2.171639295.171641295 | 0.644 | 0.042 |
| LARC | NM_015693 | chr4.128553086.128555086 | 0.653 | 0.042 |
| LARC | NM_001204183 | chr1.211847972.211849972 | 0.66 | 0.042 |
| LARC | NM_021233 | chr1.84863214.84865214 | 0.661 | 0.042 |
| LARC | NM_001085384 | chr19.58219579.58221579 | 0.662 | 0.042 |
| LARC | NM_001318967 | chr2.27592324.27594324 | 0.664 | 0.042 |
| LARC | NM_001013735 | chr9.79633570.79635570 | 1.5 | 0.042 |
| LARC | NM_001330408 | chr2.73285322.73287322 | 1.514 | 0.042 |
| LARC | NM_001136214 | chr16.69983607.69985607 | 1.533 | 0.042 |
| LARC | NM_001353356 | chr1.54664774.54666774 | 1.592 | 0.042 |
| LARC | NM_001302509 | chr16.55513444.55515444 | 1.613 | 0.042 |
| LARC | NM_001319839 | chr12.125548912.125550912 | 1.641 | 0.042 |
| LARC | NM_001330638 | chr4.6954892.6956892 | 1.653 | 0.042 |
| LARC | NM_001128174 | chr4.115518557.115520557 | 1.67 | 0.042 |
| LARC | NM_001159746 | chr17.1089616.1091616 | 1.692 | 0.042 |
| LARC | NM_001351037 | chr12.56328791.56330791 | 1.697 | 0.042 |
| LARC | NM_013353 | chr1.151147547.151149547 | 1.702 | 0.042 |
| LARC | NM_001081461 | chr17.74721881.74723881 | 1.711 | 0.042 |
| LARC | NM_152386 | chr2.223288225.223290225 | 1.716 | 0.042 |
| LARC | NM_130850 | chr14.54422609.54424609 | 1.729 | 0.042 |
| LARC | NM_032411 | chr2.106681112.106683112 | 1.731 | 0.042 |
| LARC | NM_032451 | chr16.89893906.89895906 | 1.76 | 0.042 |
| LARC | NM_130804 | chr11.64577766.64579766 | 1.767 | 0.042 |
| LARC | NM_001318383 | chr18.12253359.12255359 | 1.773 | 0.042 |
| LARC | NM_014310 | chr22.35936351.35938351 | 1.783 | 0.042 |
| LARC | NM_004953 | chr3.184037100.184039100 | 1.795 | 0.042 |
| LARC | NM_001348114 | chr13.24462743.24464743 | 1.832 | 0.042 |
| LARC | NM_031916 | chr5.10440973.10442973 | 1.836 | 0.042 |
| LARC | NM_024677 | chr4.40750913.40752913 | 1.85 | 0.042 |
| LARC | NM_012378 | chr11.124309981.124311981 | 1.857 | 0.042 |
| LARC | NM_000514 | chr5.37838782.37840782 | 1.866 | 0.042 |
| LARC | NM_006033 | chr18.47087400.47089400 | 1.905 | 0.042 |
| LARC | NM_025193 | chr16.30995518.30997518 | 1.912 | 0.042 |
| LARC | NM_006905 | chr19.43382871.43384871 | 1.916 | 0.042 |
| LARC | NM_152896 | chr9.6412150.6414150 | 1.919 | 0.042 |
| LARC | NM_198998 | chr2.241630261.241632261 | 1.938 | 0.042 |
| LARC | NM_001354325 | chr8.85094021.85096021 | 1.988 | 0.042 |
| LARC | NM_001130012 | chr16.2075868.2077868 | 1.994 | 0.042 |
| LARC | NM_012168 | chr1.11713888.11715888 | 2.003 | 0.042 |
| LARC | NM_001330355 | chr1.11713913.11715913 | 2.003 | 0.042 |
| LARC | NM_001242702 | chr10.28033778.28035778 | 2.029 | 0.042 |
| LARC | NM_001329730 | chr16.50098851.50100851 | 2.072 | 0.042 |
| LARC | NM_024103 | chr19.6458781.6460781 | 2.131 | 0.042 |
| LARC | NM_006537 | chr15.63795709.63797709 | 2.16 | 0.042 |
| LARC | NM_031466 | chr8.141467678.141469678 | 2.161 | 0.042 |
| LARC | NM_001354816 | chr4.146402485.146404485 | 2.178 | 0.042 |
| LARC | NM_052997 | chr10.37413784.37415784 | 2.198 | 0.042 |
| LARC | NM_001005366 | chr12.122017364.122019364 | 2.216 | 0.042 |
| LARC | NM_207116 | chr7.5820361.5822361 | 2.218 | 0.042 |
| LARC | NM_002518 | chr2.101435612.101437612 | 2.274 | 0.042 |
| LARC | NM_001318371 | chr22.31030801.31032801 | 2.29 | 0.042 |
| LARC | NM_015897 | chr19.4006595.4008595 | 2.396 | 0.042 |
| LARC | NM_021061 | chr8.146125846.146127846 | 2.605 | 0.042 |
| LARC | NM_000553 | chr8.30889777.30891777 | 2.615 | 0.042 |
| LARC | NM_001329933 | chr16.66913404.66915404 | 2.744 | 0.042 |
| LARC | NM_015088 | chr22.40572928.40574928 | 0.34 | 0.043 |
| LARC | NM_005019 | chr2.183386336.183388336 | 0.378 | 0.043 |
| LARC | NM_012092 | chr2.204800470.204802470 | 0.389 | 0.043 |
| LARC | NM_007178 | chr12.16034287.16036287 | 0.412 | 0.043 |
| LARC | NM_001286254 | chr6.97456951.97458951 | 0.43 | 0.043 |
| LARC | NM_001303422 | chr2.165424060.165426060 | 0.433 | 0.043 |
| LARC | NM_001105522 | chr11.89652576.89654576 | 0.437 | 0.043 |
| LARC | NM_001302777 | chr6.3117609.3119609 | 0.44 | 0.043 |
| LARC | NM_002822 | chr12.44199178.44201178 | 0.447 | 0.043 |
| LARC | NM_001253732 | chr4.159441865.159443865 | 0.45 | 0.043 |
| LARC | NM_004107 | chr19.50015435.50017435 | 0.458 | 0.043 |
| LARC | NM_001164211 | chr13.47126295.47128295 | 0.464 | 0.043 |
| LARC | NM_014963 | chr19.1173282.1175282 | 0.476 | 0.043 |
| LARC | NM_004701 | chr15.59396283.59398283 | 0.476 | 0.043 |
| LARC | NM_018166 | chr1.36788755.36790755 | 0.487 | 0.043 |
| LARC | NM_153444 | chr11.7817489.7819489 | 0.487 | 0.043 |
| LARC | NM_006420 | chr20.47537274.47539274 | 0.489 | 0.043 |
| LARC | NM_181780 | chr3.112217408.112219408 | 0.497 | 0.043 |
| LARC | NM_001004297 | chr10.45810056.45812056 | 0.498 | 0.043 |
| LARC | NM_015072 | chr14.76126550.76128550 | 0.498 | 0.043 |
| LARC | NM_003183 | chr2.9694949.9696949 | 0.505 | 0.043 |
| LARC | NM_001039697 | chr9.15421781.15423781 | 0.506 | 0.043 |
| LARC | NM_017419 | chr4.156786425.156788425 | 0.516 | 0.043 |
| LARC | NM_001170792 | chr2.38154901.38156901 | 0.524 | 0.043 |
| LARC | NM_001354871 | chr2.132438999.132440999 | 0.525 | 0.043 |
| LARC | NM_000700 | chr9.75765646.75767646 | 0.525 | 0.043 |
| LARC | NM_001348946 | chr7.87229204.87231204 | 0.525 | 0.043 |
| LARC | NM_199286 | chr12.7863049.7865049 | 0.526 | 0.043 |
| LARC | NM_001318227 | chr17.39387751.39389751 | 0.527 | 0.043 |
| LARC | NM_001321784 | chr10.5725800.5727800 | 0.527 | 0.043 |
| LARC | NM_182497 | chr17.39142387.39144387 | 0.531 | 0.043 |
| LARC | NM_000200 | chr4.70893129.70895129 | 0.532 | 0.043 |
| LARC | NM_020749 | chr8.17554246.17556246 | 0.533 | 0.043 |
| LARC | NM_001319037 | chr2.42595097.42597097 | 0.536 | 0.043 |
| LARC | NM_004531 | chr5.52404602.52406602 | 0.541 | 0.043 |
| LARC | NM_003973 | chr3.40497782.40499782 | 0.548 | 0.043 |
| LARC | NM_001258312 | chr2.183386572.183388572 | 0.548 | 0.043 |
| LARC | NM_173216 | chr3.186647314.186649314 | 0.55 | 0.043 |
| LARC | NM_153832 | chr1.168104945.168106945 | 0.556 | 0.043 |
| LARC | NM_030931 | chr20.122193.124193 | 0.556 | 0.043 |
| LARC | NM_030772 | chr1.39346298.39348298 | 0.559 | 0.043 |
| LARC | NM_023919 | chr12.10954226.10956226 | 0.561 | 0.043 |
| LARC | NM_001256794 | chr4.96011641.96013641 | 0.564 | 0.043 |
| LARC | NM_001080551 | chr9.114520813.114522813 | 0.564 | 0.043 |
| LARC | NM_001170415 | chr9.71818926.71820926 | 0.565 | 0.043 |
| LARC | NM_001199784 | chr20.1305513.1307513 | 0.565 | 0.043 |
| LARC | NM_001243078 | chr2.204570197.204572197 | 0.566 | 0.043 |
| LARC | NM_178457 | chr20.57765074.57767074 | 0.569 | 0.043 |
| LARC | NM_001284254 | chr22.24950275.24952275 | 0.569 | 0.043 |
| LARC | NM_032043 | chr17.59939920.59941920 | 0.576 | 0.043 |
| LARC | NM_001004453 | chr9.125511126.125513126 | 0.576 | 0.043 |
| LARC | NM_001206847 | chr8.30495116.30497116 | 0.58 | 0.043 |
| LARC | NM_001349778 | chr1.167904476.167906476 | 0.584 | 0.043 |
| LARC | NM_004257 | chr2.105945171.105947171 | 0.585 | 0.043 |
| LARC | NM_001005199 | chr11.56057538.56059538 | 0.59 | 0.043 |
| LARC | NM_007018 | chr9.123849573.123851573 | 0.591 | 0.043 |
| LARC | NM_001304382 | chr4.144939498.144941498 | 0.592 | 0.043 |
| LARC | NM_001289399 | chr9.2157451.2159451 | 0.593 | 0.043 |
| LARC | NM_001289400 | chr9.2157455.2159455 | 0.593 | 0.043 |
| LARC | NM_001143962 | chr1.223852436.223854436 | 0.598 | 0.043 |
| LARC | NM_001314049 | chr10.69650159.69652159 | 0.605 | 0.043 |
| LARC | NM_001347423 | chr12.9267825.9269825 | 0.61 | 0.043 |
| LARC | NM_001321936 | chr13.102969054.102971054 | 0.612 | 0.043 |
| LARC | NM_001171864 | chr10.5445793.5447793 | 0.612 | 0.043 |
| LARC | NM_001243797 | chr13.45010390.45012390 | 0.614 | 0.043 |
| LARC | NM_003117 | chr7.123564285.123566285 | 0.615 | 0.043 |
| LARC | NM_001267864 | chr14.78173413.78175413 | 0.621 | 0.043 |
| LARC | NM_001005160 | chr11.5152872.5154872 | 0.625 | 0.043 |
| LARC | NM_001080428 | chr5.167180940.167182940 | 0.626 | 0.043 |
| LARC | NM_001144764 | chr11.108407934.108409934 | 0.652 | 0.043 |
| LARC | NM_001166171 | chr9.127053249.127055249 | 1.517 | 0.043 |
| LARC | NM_002234 | chr12.5152084.5154084 | 1.524 | 0.043 |
| LARC | NM_153360 | chr20.57088994.57090994 | 1.535 | 0.043 |
| LARC | NM_001098500 | chr10.23982674.23984674 | 1.556 | 0.043 |
| LARC | NM_000728 | chr11.15094145.15096145 | 1.573 | 0.043 |
| LARC | NM_024756 | chr10.88716425.88718425 | 1.577 | 0.043 |
| LARC | NM_001319104 | chr11.133401507.133403507 | 1.584 | 0.043 |
| LARC | NM_001256812 | chr6.34077540.34079540 | 1.596 | 0.043 |
| LARC | NM_001134855 | chr1.228603583.228605583 | 1.597 | 0.043 |
| LARC | NM_173205 | chr2.113671769.113673769 | 1.598 | 0.043 |
| LARC | NM_001142633 | chr17.8868029.8870029 | 1.611 | 0.043 |
| LARC | NM_001257210 | chr5.142022866.142024866 | 1.613 | 0.043 |
| LARC | NM_001003702 | chr7.143891791.143893791 | 1.625 | 0.043 |
| LARC | NM_001256160 | chr16.10478855.10480855 | 1.628 | 0.043 |
| LARC | NM_001206747 | chr7.116138654.116140654 | 1.636 | 0.043 |
| LARC | NM_020666 | chr5.178053054.178055054 | 1.639 | 0.043 |
| LARC | NM_152450 | chr15.59729371.59731371 | 1.639 | 0.043 |
| LARC | NM_033184 | chr17.39221131.39223131 | 1.648 | 0.043 |
| LARC | NM_198989 | chr13.51417075.51419075 | 1.666 | 0.043 |
| LARC | NM_182528 | chr2.119915471.119917471 | 1.667 | 0.043 |
| LARC | NM_001101339 | chr12.25149373.25151373 | 1.686 | 0.043 |
| LARC | NM_032532 | chr6.159589428.159591428 | 1.687 | 0.043 |
| LARC | NM_025128 | chr11.65626871.65628871 | 1.7 | 0.043 |
| LARC | NM_001375 | chr19.12991335.12993335 | 1.717 | 0.043 |
| LARC | NM_207517 | chr15.84321837.84323837 | 1.722 | 0.043 |
| LARC | NM_001349113 | chr1.20125514.20127514 | 1.724 | 0.043 |
| LARC | NM_152794 | chr19.46805855.46807855 | 1.725 | 0.043 |
| LARC | NM_006695 | chr17.42384926.42386926 | 1.729 | 0.043 |
| LARC | NM_017841 | chr11.61196596.61198596 | 1.732 | 0.043 |
| LARC | NM_001305655 | chr20.62366811.62368811 | 1.732 | 0.043 |
| LARC | NM_001199877 | chr15.44068293.44070293 | 1.732 | 0.043 |
| LARC | NM_203423 | chr4.7939727.7941727 | 1.744 | 0.043 |
| LARC | NM_003076 | chr12.50477982.50479982 | 1.746 | 0.043 |
| LARC | NM_033510 | chr15.40649435.40651435 | 1.748 | 0.043 |
| LARC | NM_001286526 | chr16.30904623.30906623 | 1.753 | 0.043 |
| LARC | NM_001351263 | chr10.71210222.71212222 | 1.763 | 0.043 |
| LARC | NM_001136002 | chr7.123672523.123674523 | 1.77 | 0.043 |
| LARC | NM_001322841 | chr17.1131974.1133974 | 1.782 | 0.043 |
| LARC | NM_021570 | chr9.96716608.96718608 | 1.783 | 0.043 |
| LARC | NM_001351035 | chr12.56328617.56330617 | 1.799 | 0.043 |
| LARC | NM_001137560 | chr6.44237479.44239479 | 1.801 | 0.043 |
| LARC | NM_018134 | chr1.32670235.32672235 | 1.808 | 0.043 |
| LARC | NM_001300797 | chr12.131355538.131357538 | 1.816 | 0.043 |
| LARC | NM_001288662 | chr4.5888540.5890540 | 1.822 | 0.043 |
| LARC | NM_004864 | chr19.18495769.18497769 | 1.837 | 0.043 |
| LARC | NM_001141979 | chr15.43784369.43786369 | 1.84 | 0.043 |
| LARC | NM_001127222 | chr19.13616274.13618274 | 1.846 | 0.043 |
| LARC | NM_005576 | chr15.74217798.74219798 | 1.847 | 0.043 |
| LARC | NM_205850 | chr15.48412168.48414168 | 1.854 | 0.043 |
| LARC | NM_001282988 | chr14.24421943.24423943 | 1.858 | 0.043 |
| LARC | NM_015597 | chr9.139220931.139222931 | 1.865 | 0.043 |
| LARC | NM_001320419 | chr2.99796524.99798524 | 1.891 | 0.043 |
| LARC | NM_145212 | chr2.99796541.99798541 | 1.891 | 0.043 |
| LARC | NM_001145785 | chr19.19280098.19282098 | 1.898 | 0.043 |
| LARC | NM_006026 | chr3.129034120.129036120 | 1.947 | 0.043 |
| LARC | NM_005541 | chr2.233923676.233925676 | 1.947 | 0.043 |
| LARC | NM_001288767 | chr16.31468593.31470593 | 1.948 | 0.043 |
| LARC | NM_001164463 | chr2.113190222.113192222 | 1.959 | 0.043 |
| LARC | NM_001330625 | chr4.4290923.4292923 | 1.965 | 0.043 |
| LARC | NM_001193524 | chr16.67570364.67572364 | 1.987 | 0.043 |
| LARC | NM_001349962 | chr10.101418133.101420133 | 1.993 | 0.043 |
| LARC | NM_001135816 | chr13.24462577.24464577 | 2.025 | 0.043 |
| LARC | NM_152412 | chr8.125984538.125986538 | 2.04 | 0.043 |
| LARC | NM_006459 | chr10.101944734.101946734 | 2.043 | 0.043 |
| LARC | NM_001270726 | chr22.18120349.18122349 | 2.053 | 0.043 |
| LARC | NM_016504 | chr17.48449562.48451562 | 2.053 | 0.043 |
| LARC | NM_001166131 | chr17.48449580.48451580 | 2.053 | 0.043 |
| LARC | NM_019891 | chr1.236444339.236446339 | 2.09 | 0.043 |
| LARC | NM_001277224 | chr1.159892507.159894507 | 2.122 | 0.043 |
| LARC | NM_001300721 | chr11.45943226.45945226 | 2.139 | 0.043 |
| LARC | NM_001330988 | chr9.130829478.130831478 | 2.139 | 0.043 |
| LARC | NM_004270 | chr5.156568921.156570921 | 2.144 | 0.043 |
| LARC | NM_001195605 | chr19.56123958.56125958 | 2.153 | 0.043 |
| LARC | NM_001302621 | chr7.100492592.100494592 | 2.157 | 0.043 |
| LARC | NM_001743 | chr2.47402762.47404762 | 2.195 | 0.043 |
| LARC | NM_018192 | chr3.189837908.189839908 | 2.227 | 0.043 |
| LARC | NM_002775 | chr10.124220040.124222040 | 2.281 | 0.043 |
| LARC | NM_015933 | chr3.48480647.48482647 | 2.291 | 0.043 |
| LARC | NM_003966 | chr5.9545233.9547233 | 2.333 | 0.043 |
| LARC | NM_138718 | chr6.35991277.35993277 | 2.343 | 0.043 |
| LARC | NM_005239 | chr21.40176754.40178754 | 2.397 | 0.043 |
| LARC | NM_198723 | chr20.62687324.62689324 | 2.453 | 0.043 |
| LARC | NM_152652 | chr16.30405739.30407739 | 2.465 | 0.043 |
| LARC | NM_001301647 | chr4.170946581.170948581 | 2.477 | 0.043 |
| LARC | NM_001159287 | chr12.6975583.6977583 | 2.573 | 0.043 |
| LARC | NM_001350110 | chr1.155948081.155950081 | 2.574 | 0.043 |
| LARC | NM_001114600 | chr1.16692524.16694524 | 2.621 | 0.043 |
| LARC | NM_001329929 | chr16.66913263.66915263 | 2.779 | 0.043 |
| LARC | NM_001258311 | chr1.230560674.230562674 | 2.828 | 0.043 |
| LARC | NM_001010874 | chr4.65274178.65276178 | 0.345 | 0.044 |
| LARC | NM_024416 | chr9.95165981.95167981 | 0.42 | 0.044 |
| LARC | NM_014495 | chr1.63062157.63064157 | 0.449 | 0.044 |
| LARC | NM_032440 | chr10.98591711.98593711 | 1.974 | 0.044 |
| LARC | NM_198124 | chr8.114388382.114390382 | 0.291 | 0.045 |
| LARC | NM_205864 | chr6.7388942.7390942 | 0.374 | 0.045 |
| LARC | NM_001297429 | chr13.42711177.42713177 | 0.39 | 0.045 |
| LARC | NM_001207004 | chr4.70145216.70147216 | 0.399 | 0.045 |
| LARC | NM_014715 | chr11.128893088.128895088 | 0.42 | 0.045 |
| LARC | NM_001242916 | chr15.80363902.80365902 | 0.422 | 0.045 |
| LARC | NM_022160 | chr9.22445839.22447839 | 0.426 | 0.045 |
| LARC | NM_001323563 | chr20.5985718.5987718 | 0.435 | 0.045 |
| LARC | NM_001307955 | chr18.52253989.52255989 | 0.445 | 0.045 |
| LARC | NM_021201 | chr11.60144957.60146957 | 0.447 | 0.045 |
| LARC | NM_020872 | chr3.74569291.74571291 | 0.449 | 0.045 |
| LARC | NM_001131010 | chr3.18479265.18481265 | 0.449 | 0.045 |
| LARC | NM_001317103 | chr1.78469511.78471511 | 0.464 | 0.045 |
| LARC | NM_013373 | chr22.20118363.20120363 | 0.47 | 0.045 |
| LARC | NM_001205220 | chr6.49843809.49845809 | 0.472 | 0.045 |
| LARC | NM_182724 | chr10.35483829.35485829 | 0.48 | 0.045 |
| LARC | NM_001252156 | chr5.154316776.154318776 | 0.487 | 0.045 |
| LARC | NM_145080 | chr16.27279113.27281113 | 0.494 | 0.045 |
| LARC | NM_001682 | chr12.90048844.90050844 | 0.495 | 0.045 |
| LARC | NM_001206482 | chr7.37392272.37394272 | 0.498 | 0.045 |
| LARC | NM_000231 | chr13.23754059.23756059 | 0.499 | 0.045 |
| LARC | NM_001348242 | chr21.47087787.47089787 | 0.499 | 0.045 |
| LARC | NM_001270679 | chr10.74450888.74452888 | 0.502 | 0.045 |
| LARC | NM_002543 | chr12.10323790.10325790 | 0.503 | 0.045 |
| LARC | NM_003145 | chr1.155989758.155991758 | 0.503 | 0.045 |
| LARC | NM_031423 | chr1.163290722.163292722 | 0.515 | 0.045 |
| LARC | NM_152586 | chr10.75334433.75336433 | 0.518 | 0.045 |
| LARC | NM_001243429 | chr21.39955869.39957869 | 0.521 | 0.045 |
| LARC | NM_001164443 | chr5.74531703.74533703 | 0.527 | 0.045 |
| LARC | NM_001145526 | chr17.76182397.76184397 | 0.532 | 0.045 |
| LARC | NM_014519 | chr17.5014569.5016569 | 0.537 | 0.045 |
| LARC | NM_001319655 | chr12.76815872.76817872 | 0.537 | 0.045 |
| LARC | NM_145341 | chr10.112630552.112632552 | 0.538 | 0.045 |
| LARC | NM_006421 | chr8.68254912.68256912 | 0.538 | 0.045 |
| LARC | NM_004029 | chr11.614999.616999 | 0.538 | 0.045 |
| LARC | NM_001199241 | chr2.143634194.143636194 | 0.539 | 0.045 |
| LARC | NM_152489 | chr1.64668300.64670300 | 0.544 | 0.045 |
| LARC | NM_012351 | chr1.159408511.159410511 | 0.544 | 0.045 |
| LARC | NM_001303120 | chr21.31913183.31915183 | 0.544 | 0.045 |
| LARC | NM_181690 | chr1.244005584.244007584 | 0.548 | 0.045 |
| LARC | NM_002006 | chr4.123746862.123748862 | 0.549 | 0.045 |
| LARC | NM_001737 | chr5.39363662.39365662 | 0.55 | 0.045 |
| LARC | NM_001100917 | chr12.85429055.85431055 | 0.553 | 0.045 |
| LARC | NM_003513 | chr6.26032796.26034796 | 0.554 | 0.045 |
| LARC | NM_001300866 | chr6.76202630.76204630 | 0.563 | 0.045 |
| LARC | NM_001330605 | chr18.53068208.53070208 | 0.569 | 0.045 |
| LARC | NM_001018080 | chr11.30251562.30253562 | 0.57 | 0.045 |
| LARC | NM_001126117 | chr17.7577811.7579811 | 0.574 | 0.045 |
| LARC | NM_001290300 | chr3.25381292.25383292 | 0.575 | 0.045 |
| LARC | NM_000667 | chr4.100211185.100213185 | 0.576 | 0.045 |
| LARC | NM_002313 | chr10.116417058.116419058 | 0.578 | 0.045 |
| LARC | NM_006419 | chr4.78431906.78433906 | 0.581 | 0.045 |
| LARC | NM_005824 | chr7.102552343.102554343 | 0.583 | 0.045 |
| LARC | NM_144634 | chr3.42451094.42453094 | 0.583 | 0.045 |
| LARC | NM_025153 | chr5.160278219.160280219 | 0.585 | 0.045 |
| LARC | NM_005795 | chr2.188312021.188314021 | 0.585 | 0.045 |
| LARC | NM_001318040 | chr9.116637561.116639561 | 0.586 | 0.045 |
| LARC | NM_001373 | chr1.225116355.225118355 | 0.594 | 0.045 |
| LARC | NM_130767 | chr5.80688988.80690988 | 0.597 | 0.045 |
| LARC | NM_005212 | chr4.71107332.71109332 | 0.597 | 0.045 |
| LARC | NM_032572 | chr14.21509384.21511384 | 0.601 | 0.045 |
| LARC | NM_020056 | chr6.32708162.32710162 | 0.603 | 0.045 |
| LARC | NM_152392 | chr2.61403552.61405552 | 0.603 | 0.045 |
| LARC | NM_001005327 | chr1.158686905.158688905 | 0.607 | 0.045 |
| LARC | NM_001077244 | chr11.105479799.105481799 | 0.608 | 0.045 |
| LARC | NM_172365 | chr14.65015619.65017619 | 0.61 | 0.045 |
| LARC | NM_207393 | chr19.46626931.46628931 | 0.614 | 0.045 |
| LARC | NM_144625 | chr1.241814579.241816579 | 0.614 | 0.045 |
| LARC | NM_078488 | chr6.133083598.133085598 | 0.615 | 0.045 |
| LARC | NM_001244638 | chr10.63807969.63809969 | 0.616 | 0.045 |
| LARC | NM_178127 | chr11.101786253.101788253 | 0.616 | 0.045 |
| LARC | NM_001329524 | chr19.37568123.37570123 | 0.622 | 0.045 |
| LARC | NM_001042521 | chr2.191001485.191003485 | 0.624 | 0.045 |
| LARC | NM_024058 | chr13.44734393.44736393 | 0.625 | 0.045 |
| LARC | NM_024637 | chr7.99765373.99767373 | 0.631 | 0.045 |
| LARC | NM_031935 | chr1.185702682.185704682 | 0.632 | 0.045 |
| LARC | NM_001191049 | chr4.83811433.83813433 | 0.636 | 0.045 |
| LARC | NM_001309516 | chr2.209223568.209225568 | 0.638 | 0.045 |
| LARC | NM_014364 | chr19.36023313.36025313 | 0.643 | 0.045 |
| LARC | NM_003975 | chr1.156785640.156787640 | 0.644 | 0.045 |
| LARC | NM_001282878 | chr1.203737765.203739765 | 0.649 | 0.045 |
| LARC | NM_144602 | chr16.49406718.49408718 | 0.649 | 0.045 |
| LARC | NM_001144756 | chr4.72903848.72905848 | 0.652 | 0.045 |
| LARC | NM_001349800 | chr1.200635068.200637068 | 0.652 | 0.045 |
| LARC | NM_001005338 | chr3.97850541.97852541 | 0.656 | 0.045 |
| LARC | NM_018936 | chr5.140473190.140475190 | 0.658 | 0.045 |
| LARC | NM_014407 | chr3.178968403.178970403 | 0.658 | 0.045 |
| LARC | NM_001354211 | chr4.114899878.114901878 | 0.66 | 0.045 |
| LARC | NM_001350717 | chr7.15013402.15015402 | 0.663 | 0.045 |
| LARC | NM_001270876 | chr1.37979420.37981420 | 0.665 | 0.045 |
| LARC | NM_147130 | chr6.31559762.31561762 | 1.511 | 0.045 |
| LARC | NM_019113 | chr19.49257815.49259815 | 1.531 | 0.045 |
| LARC | NM_178120 | chr2.172949207.172951207 | 1.532 | 0.045 |
| LARC | NM_001307985 | chr11.64322072.64324072 | 1.561 | 0.045 |
| LARC | NM_001142459 | chr7.150883478.150885478 | 1.568 | 0.045 |
| LARC | NM_001081955 | chr17.63132455.63134455 | 1.574 | 0.045 |
| LARC | NM_152413 | chr8.37796647.37798647 | 1.58 | 0.045 |
| LARC | NM_001099782 | chr22.24640110.24642110 | 1.58 | 0.045 |
| LARC | NM_001161427 | chr19.52838497.52840497 | 1.586 | 0.045 |
| LARC | NM_001271197 | chr16.69983888.69985888 | 1.605 | 0.045 |
| LARC | NM_020313 | chr16.57480440.57482440 | 1.607 | 0.045 |
| LARC | NM_175881 | chr15.76015318.76017318 | 1.628 | 0.045 |
| LARC | NM_182625 | chr2.17934124.17936124 | 1.639 | 0.045 |
| LARC | NM_001288 | chr6.31703341.31705341 | 1.641 | 0.045 |
| LARC | NM_001320628 | chr16.29464526.29466526 | 1.644 | 0.045 |
| LARC | NM_001297420 | chr12.1041242.1043242 | 1.647 | 0.045 |
| LARC | NM_001321041 | chr15.90038814.90040814 | 1.65 | 0.045 |
| LARC | NM_000476 | chr9.130639022.130641022 | 1.653 | 0.045 |
| LARC | NM_001105565 | chr11.57309113.57311113 | 1.659 | 0.045 |
| LARC | NM_080871 | chr7.150883919.150885919 | 1.668 | 0.045 |
| LARC | NM_014716 | chr17.7238847.7240847 | 1.676 | 0.045 |
| LARC | NM_001736 | chr19.47812103.47814103 | 1.681 | 0.045 |
| LARC | NM_001537 | chr16.83840507.83842507 | 1.706 | 0.045 |
| LARC | NM_001355229 | chr9.99774964.99776964 | 1.708 | 0.045 |
| LARC | NM_001351079 | chr10.116163537.116165537 | 1.751 | 0.045 |
| LARC | NM_001305020 | chr16.30545291.30547291 | 1.763 | 0.045 |
| LARC | NM_025165 | chr15.44068502.44070502 | 1.764 | 0.045 |
| LARC | NM_020992 | chr10.97049905.97051905 | 1.765 | 0.045 |
| LARC | NM_001303029 | chr11.391598.393598 | 1.773 | 0.045 |
| LARC | NM_001080398 | chr9.114246025.114248025 | 1.776 | 0.045 |
| LARC | NM_017816 | chr4.4290896.4292896 | 1.778 | 0.045 |
| LARC | NM_003936 | chr2.219823349.219825349 | 1.786 | 0.045 |
| LARC | NM_001319041 | chr6.123099619.123101619 | 1.804 | 0.045 |
| LARC | NM_004447 | chr12.15941510.15943510 | 1.806 | 0.045 |
| LARC | NM_148962 | chr2.42990401.42992401 | 1.813 | 0.045 |
| LARC | NM_000097 | chr3.98311455.98313455 | 1.818 | 0.045 |
| LARC | NM_001304509 | chr9.116138289.116140289 | 1.825 | 0.045 |
| LARC | NM_001300984 | chr15.28343458.28345458 | 1.827 | 0.045 |
| LARC | NM_001318162 | chr7.99035998.99037998 | 1.828 | 0.045 |
| LARC | NM_021064 | chr6.27099816.27101816 | 1.844 | 0.045 |
| LARC | NM_021133 | chr1.182557394.182559394 | 1.866 | 0.045 |
| LARC | NM_001292018 | chr17.76718832.76720832 | 1.906 | 0.045 |
| LARC | NM_001319307 | chr4.128885410.128887410 | 1.91 | 0.045 |
| LARC | NM_001324494 | chr16.30405432.30407432 | 1.911 | 0.045 |
| LARC | NM_012284 | chr12.49931939.49933939 | 1.946 | 0.045 |
| LARC | NM_004786 | chr18.54304920.54306920 | 1.95 | 0.045 |
| LARC | NM_020191 | chr3.139061860.139063860 | 1.972 | 0.045 |
| LARC | NM_138353 | chr19.14062285.14064285 | 1.982 | 0.045 |
| LARC | NM_001018137 | chr17.49241795.49243795 | 2.017 | 0.045 |
| LARC | NM_080611 | chr20.30457547.30459547 | 2.021 | 0.045 |
| LARC | NM_178123 | chr2.180128350.180130350 | 2.088 | 0.045 |
| LARC | NM_014165 | chr6.97344767.97346767 | 2.106 | 0.045 |
| LARC | NM_032211 | chr10.100027007.100029007 | 2.126 | 0.045 |
| LARC | NM_001321185 | chr17.76123875.76125875 | 2.146 | 0.045 |
| LARC | NM_014268 | chr18.32620323.32622323 | 2.161 | 0.045 |
| LARC | NM_001010908 | chr10.16563004.16565004 | 2.175 | 0.045 |
| LARC | NM_001321906 | chr2.115918509.115920509 | 2.218 | 0.045 |
| LARC | NM_001178034 | chr2.115918512.115920512 | 2.218 | 0.045 |
| LARC | NM_001301201 | chr20.360938.362938 | 2.219 | 0.045 |
| LARC | NM_001328653 | chr1.46597380.46599380 | 2.228 | 0.045 |
| LARC | NM_001302813 | chr20.58629979.58631979 | 2.229 | 0.045 |
| LARC | NM_002341 | chr6.31549202.31551202 | 2.256 | 0.045 |
| LARC | NM_001198682 | chr17.49242632.49244632 | 2.264 | 0.045 |
| LARC | NM_001317803 | chr7.75622992.75624992 | 2.292 | 0.045 |
| LARC | NM_001100624 | chr16.81039102.81041102 | 2.335 | 0.045 |
| LARC | NM_001318085 | chr4.110353870.110355870 | 2.373 | 0.045 |
| LARC | NM_005186 | chr11.64948303.64950303 | 2.405 | 0.045 |
| LARC | NM_000095 | chr19.18901114.18903114 | 2.412 | 0.045 |
| LARC | NM_001284308 | chr7.994043.996043 | 2.689 | 0.045 |
| LARC | NM_015285 | chr18.54317615.54319615 | 2.729 | 0.045 |
| LARC | NM_001271998 | chr1.229405808.229407808 | 7.891 | 0.045 |
| LARC | NM_001130069 | chr7.56130916.56132916 | 0.371 | 0.045 |
| LARC | NM_016200 | chr7.117823085.117825085 | 0.409 | 0.045 |
| LARC | NM_001199179 | chr3.130611834.130613834 | 0.43 | 0.045 |
| LARC | NM_005049 | chr21.45526207.45528207 | 0.463 | 0.045 |
| LARC | NM_001527 | chr6.114291359.114293359 | 0.467 | 0.045 |
| LARC | NM_001193466 | chr17.44269166.44271166 | 0.489 | 0.045 |
| LARC | NM_002922 | chr1.192543856.192545856 | 0.494 | 0.045 |
| LARC | NM_001199837 | chr7.26399503.26401503 | 0.504 | 0.045 |
| LARC | NM_181558 | chr13.34391205.34393205 | 0.509 | 0.045 |
| LARC | NM_001289003 | chr16.71748743.71750743 | 0.511 | 0.045 |
| LARC | NM_001122757 | chr3.87324737.87326737 | 0.521 | 0.045 |
| LARC | NM_207040 | chr15.57510654.57512654 | 0.53 | 0.045 |
| LARC | NM_001242840 | chr1.228326928.228328928 | 0.538 | 0.045 |
| LARC | NM_001257268 | chr14.104407645.104409645 | 0.542 | 0.045 |
| LARC | NM_001012710 | chr11.71275608.71277608 | 0.545 | 0.045 |
| LARC | NM_001318754 | chr6.55955459.55957459 | 0.545 | 0.045 |
| LARC | NM_006944 | chr2.234958345.234960345 | 0.547 | 0.045 |
| LARC | NM_001287140 | chr8.80992066.80994066 | 0.553 | 0.045 |
| LARC | NM_001267723 | chr19.46497718.46499718 | 0.567 | 0.045 |
| LARC | NM_001198615 | chr6.136787013.136789013 | 0.567 | 0.045 |
| LARC | NM_001282293 | chr7.150724508.150726508 | 0.598 | 0.045 |
| LARC | NM_006432 | chr14.74959084.74961084 | 0.601 | 0.045 |
| LARC | NM_001130071 | chr19.56186990.56188990 | 0.604 | 0.045 |
| LARC | NM_001278548 | chr10.49608654.49610654 | 0.608 | 0.045 |
| LARC | NM_001204307 | chr4.72668758.72670758 | 0.635 | 0.045 |
| LARC | NM_001085420 | chr3.146323003.146325003 | 0.647 | 0.045 |
| LARC | NM_001256597 | chr8.49983899.49985899 | 0.651 | 0.045 |
| LARC | NM_001321315 | chr2.191522883.191524883 | 0.666 | 0.045 |
| LARC | NM_152778 | chr4.128886139.128888139 | 1.577 | 0.045 |
| LARC | NM_003353 | chr2.27530313.27532313 | 1.679 | 0.045 |
| LARC | NM_030811 | chr20.3025674.3027674 | 1.688 | 0.045 |
| LARC | NM_014431 | chr10.72237563.72239563 | 1.689 | 0.045 |
| LARC | NM_001109763 | chr16.28073830.28075830 | 1.712 | 0.045 |
| LARC | NM_005054 | chr2.113190107.113192107 | 1.783 | 0.045 |
| LARC | NM_007215 | chr17.62492184.62494184 | 1.877 | 0.045 |
| LARC | NM_001320479 | chr20.30457467.30459467 | 1.922 | 0.045 |
| LARC | NM_003626 | chr11.70115805.70117805 | 1.934 | 0.045 |
| LARC | NM_001256264 | chr17.18160870.18162870 | 1.943 | 0.045 |
| LARC | NM_139014 | chr6.35994453.35996453 | 1.97 | 0.045 |
| LARC | NM_024519 | chr16.67561716.67563716 | 1.989 | 0.045 |
| LARC | NM_001193522 | chr16.67561719.67563719 | 1.989 | 0.045 |
| LARC | NM_006242 | chr20.58514352.58516352 | 2.009 | 0.045 |
| LARC | NM_003190 | chr6.33281164.33283164 | 2.015 | 0.045 |
| LARC | NM_199073 | chr3.49057617.49059617 | 2.065 | 0.045 |
| LARC | NM_002077 | chr9.127702386.127704386 | 2.122 | 0.045 |
| LARC | NM_002949 | chr17.79669399.79671399 | 2.124 | 0.045 |
| LARC | NM_022092 | chr16.837621.839621 | 2.134 | 0.045 |
| LARC | NM_001301188 | chr20.360949.362949 | 2.134 | 0.045 |
| LARC | NM_020698 | chr12.95043338.95045338 | 2.17 | 0.045 |
| LARC | NM_014811 | chr9.138370647.138372647 | 2.202 | 0.045 |
| LARC | NM_001287249 | chr2.242672993.242674993 | 2.233 | 0.045 |
| LARC | NM_015567 | chr13.88323869.88325869 | 2.295 | 0.045 |
| LARC | NM_001330161 | chr2.242625426.242627426 | 2.367 | 0.045 |
| LARC | NM_004327 | chr22.23521551.23523551 | 2.476 | 0.045 |
| LARC | NM_005349 | chr4.26320331.26322331 | 2.548 | 0.045 |
| LARC | NM_145315 | chr6.108615077.108617077 | 2.578 | 0.045 |
| LARC | NM_001317090 | chr11.64058481.64060481 | 2.758 | 0.045 |
| LARC | NM_016304 | chr15.55488231.55490231 | 0.358 | 0.046 |
| LARC | NM_001197317 | chr12.9821303.9823303 | 0.416 | 0.046 |
| LARC | NM_201434 | chr17.40306062.40308062 | 0.419 | 0.046 |
| LARC | NM_022122 | chr11.102575468.102577468 | 0.427 | 0.046 |
| LARC | NM_001348 | chr19.3970121.3972121 | 0.433 | 0.046 |
| LARC | NM_001253388 | chr3.360365.362365 | 0.438 | 0.046 |
| LARC | NM_001270993 | chr4.75173186.75175186 | 0.443 | 0.046 |
| LARC | NM_001308193 | chr5.173319053.173321053 | 0.453 | 0.046 |
| LARC | NM_001316352 | chr11.13516722.13518722 | 0.46 | 0.046 |
| LARC | NM_033306 | chr11.104826422.104828422 | 0.465 | 0.046 |
| LARC | NM_004133 | chr8.76451202.76453202 | 0.466 | 0.046 |
| LARC | NM_001286523 | chr21.15917681.15919681 | 0.468 | 0.046 |
| LARC | NM_001291339 | chr8.67995082.67997082 | 0.486 | 0.046 |
| LARC | NM_000493 | chr6.116446296.116448296 | 0.492 | 0.046 |
| LARC | NM_005420 | chr4.70724870.70726870 | 0.494 | 0.046 |
| LARC | NM_002896 | chr11.66405087.66407087 | 0.499 | 0.046 |
| LARC | NM_001172651 | chr19.9485991.9487991 | 0.501 | 0.046 |
| LARC | NM_001288811 | chr17.45119079.45121079 | 0.507 | 0.046 |
| LARC | NM_001286803 | chr9.132387431.132389431 | 0.508 | 0.046 |
| LARC | NM_001321913 | chr2.116100392.116102392 | 0.516 | 0.046 |
| LARC | NM_020529 | chr14.35872960.35874960 | 0.523 | 0.046 |
| LARC | NM_004967 | chr4.88719701.88721701 | 0.523 | 0.046 |
| LARC | NM_017855 | chr4.71061243.71063243 | 0.527 | 0.046 |
| LARC | NM_001308020 | chr18.39534162.39536162 | 0.528 | 0.046 |
| LARC | NM_001323677 | chr10.31609549.31611549 | 0.531 | 0.046 |
| LARC | NM_001303049 | chr18.3251210.3253210 | 0.533 | 0.046 |
| LARC | NM_001142733 | chr3.57325710.57327710 | 0.536 | 0.046 |
| LARC | NM_004467 | chr8.17752047.17754047 | 0.538 | 0.046 |
| LARC | NM_001199805 | chr12.10561745.10563745 | 0.538 | 0.046 |
| LARC | NM_002603 | chr8.66700329.66702329 | 0.539 | 0.046 |
| LARC | NM_012338 | chr7.120497177.120499177 | 0.54 | 0.046 |
| LARC | NM_001242946 | chr7.37959162.37961162 | 0.54 | 0.046 |
| LARC | NM_021923 | chr4.1005251.1007251 | 0.542 | 0.046 |
| LARC | NM_000386 | chr17.28618184.28620184 | 0.543 | 0.046 |
| LARC | NM_144595 | chr13.78314294.78316294 | 0.543 | 0.046 |
| LARC | NM_052831 | chr6.133118747.133120747 | 0.544 | 0.046 |
| LARC | NM_001354589 | chr3.61236135.61238135 | 0.548 | 0.046 |
| LARC | NM_033554 | chr6.33040454.33042454 | 0.548 | 0.046 |
| LARC | NM_000942 | chr15.64454354.64456354 | 0.551 | 0.046 |
| LARC | NM_213658 | chr12.10606215.10608215 | 0.551 | 0.046 |
| LARC | NM_001306219 | chr15.57510627.57512627 | 0.552 | 0.046 |
| LARC | NM_001324120 | chr15.22891218.22893218 | 0.553 | 0.046 |
| LARC | NM_001319242 | chr12.10162225.10164225 | 0.555 | 0.046 |
| LARC | NM_001037668 | chr8.7352367.7354367 | 0.557 | 0.046 |
| LARC | NM_000587 | chr5.40908353.40910353 | 0.558 | 0.046 |
| LARC | NM_003021 | chr19.2782354.2784354 | 0.559 | 0.046 |
| LARC | NM_031952 | chr5.95017714.95019714 | 0.559 | 0.046 |
| LARC | NM_001288997 | chr4.83930987.83932987 | 0.561 | 0.046 |
| LARC | NM_001252271 | chr1.93341707.93343707 | 0.561 | 0.046 |
| LARC | NM_001312688 | chr1.107681539.107683539 | 0.562 | 0.046 |
| LARC | NM_013309 | chr15.45814002.45816002 | 0.563 | 0.046 |
| LARC | NM_001109809 | chr6.29643931.29645931 | 0.563 | 0.046 |
| LARC | NM_147203 | chr8.17751913.17753913 | 0.564 | 0.046 |
| LARC | NM_001290047 | chr22.17955627.17957627 | 0.567 | 0.046 |
| LARC | NM_024642 | chr9.101568980.101570980 | 0.569 | 0.046 |
| LARC | NM_001314048 | chr9.6214785.6216785 | 0.569 | 0.046 |
| LARC | NM_001304448 | chr12.10606284.10608284 | 0.572 | 0.046 |
| LARC | NM_016161 | chr3.137850229.137852229 | 0.575 | 0.046 |
| LARC | NM_001206769 | chr11.84027382.84029382 | 0.577 | 0.046 |
| LARC | NM_003068 | chr8.49832999.49834999 | 0.577 | 0.046 |
| LARC | NM_032981 | chr18.32397301.32399301 | 0.579 | 0.046 |
| LARC | NM_001351284 | chr12.86649196.86651196 | 0.581 | 0.046 |
| LARC | NM_001139510 | chr6.127662552.127664552 | 0.581 | 0.046 |
| LARC | NM_017418 | chr9.117903096.117905096 | 0.583 | 0.046 |
| LARC | NM_001324124 | chr15.22891613.22893613 | 0.585 | 0.046 |
| LARC | NM_001002010 | chr7.33101433.33103433 | 0.587 | 0.046 |
| LARC | NM_001130089 | chr16.75680585.75682585 | 0.589 | 0.046 |
| LARC | NM_016729 | chr11.71902172.71904172 | 0.592 | 0.046 |
| LARC | NM_005684 | chr1.174416211.174418211 | 0.592 | 0.046 |
| LARC | NM_019897 | chr9.35957151.35959151 | 0.593 | 0.046 |
| LARC | NM_001135649 | chr2.88751053.88753053 | 0.594 | 0.046 |
| LARC | NM_001004691 | chr1.248486870.248488870 | 0.594 | 0.046 |
| LARC | NM_001039580 | chr4.156297122.156299122 | 0.596 | 0.046 |
| LARC | NM_005142 | chr11.59611974.59613974 | 0.596 | 0.046 |
| LARC | NM_001963 | chr4.110833039.110835039 | 0.602 | 0.046 |
| LARC | NM_001198532 | chr8.107459151.107461151 | 0.603 | 0.046 |
| LARC | NM_001256596 | chr8.49965894.49967894 | 0.603 | 0.046 |
| LARC | NM_001198942 | chr18.32397246.32399246 | 0.604 | 0.046 |
| LARC | NM_001073 | chr4.70079449.70081449 | 0.605 | 0.046 |
| LARC | NM_001005487 | chr1.247835343.247837343 | 0.607 | 0.046 |
| LARC | NM_006219 | chr3.138477201.138479201 | 0.611 | 0.046 |
| LARC | NM_001145417 | chr3.135912310.135914310 | 0.612 | 0.046 |
| LARC | NM_001145095 | chr8.133116512.133118512 | 0.616 | 0.046 |
| LARC | NM_030589 | chr19.41387657.41389657 | 0.621 | 0.046 |
| LARC | NM_198827 | chr12.131437451.131439451 | 0.623 | 0.046 |
| LARC | NM_014208 | chr4.88528680.88530680 | 0.631 | 0.046 |
| LARC | NM_001509 | chr6.28492788.28494788 | 0.632 | 0.046 |
| LARC | NM_001164342 | chr3.114101489.114103489 | 0.634 | 0.046 |
| LARC | NM_001312689 | chr10.28271035.28273035 | 0.637 | 0.046 |
| LARC | NM_001300792 | chr5.132072270.132074270 | 0.638 | 0.046 |
| LARC | NM_001318864 | chr3.57203345.57205345 | 0.638 | 0.046 |
| LARC | NM_024015 | chr17.46654743.46656743 | 0.639 | 0.046 |
| LARC | NM_001145054 | chr2.74643844.74645844 | 0.64 | 0.046 |
| LARC | NM_002427 | chr11.102825463.102827463 | 0.647 | 0.046 |
| LARC | NM_001775 | chr4.15778887.15780887 | 0.649 | 0.046 |
| LARC | NM_145007 | chr19.56347128.56349128 | 0.65 | 0.046 |
| LARC | NM_001190810 | chr10.47212626.47214626 | 0.652 | 0.046 |
| LARC | NM_001807 | chr9.135936364.135938364 | 0.655 | 0.046 |
| LARC | NM_001256488 | chr3.121467614.121469614 | 0.658 | 0.046 |
| LARC | NM_021957 | chr12.21756781.21758781 | 0.659 | 0.046 |
| LARC | NM_000130 | chr1.169554769.169556769 | 0.659 | 0.046 |
| LARC | NM_001306142 | chr4.8872543.8874543 | 0.662 | 0.046 |
| LARC | NM_152272 | chr8.23100149.23102149 | 0.664 | 0.046 |
| LARC | NM_001145861 | chr8.99128520.99130520 | 0.664 | 0.046 |
| LARC | NM_020167 | chr5.151783840.151785840 | 0.665 | 0.046 |
| LARC | NM_001355235 | chr1.248096067.248098067 | 0.666 | 0.046 |
| LARC | NM_001313990 | chr2.217523340.217525340 | 1.51 | 0.046 |
| LARC | NM_005047 | chr9.123604299.123606299 | 1.51 | 0.046 |
| LARC | NM_173515 | chr6.154830793.154832793 | 1.52 | 0.046 |
| LARC | NM_001137671 | chr18.14542599.14544599 | 1.522 | 0.046 |
| LARC | NM_000702 | chr1.160084519.160086519 | 1.524 | 0.046 |
| LARC | NM_001353069 | chr9.124029379.124031379 | 1.526 | 0.046 |
| LARC | NM_001284305 | chr22.47168823.47170823 | 1.541 | 0.046 |
| LARC | NM_003244 | chr18.3449169.3451169 | 1.553 | 0.046 |
| LARC | NM_001258282 | chr9.29212965.29214965 | 1.556 | 0.046 |
| LARC | NM_001355251 | chr2.111001997.111003997 | 1.575 | 0.046 |
| LARC | NM_001276260 | chr9.116297756.116299756 | 1.586 | 0.046 |
| LARC | NM_001172813 | chr8.117962189.117964189 | 1.588 | 0.046 |
| LARC | NM_004844 | chr3.15373136.15375136 | 1.598 | 0.046 |
| LARC | NM_003323 | chr19.49400996.49402996 | 1.628 | 0.046 |
| LARC | NM_001318793 | chr4.154604403.154606403 | 1.629 | 0.046 |
| LARC | NM_001053 | chr16.1127780.1129780 | 1.635 | 0.046 |
| LARC | NM_144732 | chr19.41767380.41769380 | 1.636 | 0.046 |
| LARC | NM_198555 | chr2.97777889.97779889 | 1.636 | 0.046 |
| LARC | NM_001235 | chr11.75272100.75274100 | 1.638 | 0.046 |
| LARC | NM_000595 | chr6.31539070.31541070 | 1.639 | 0.046 |
| LARC | NM_001346858 | chr22.26907472.26909472 | 1.639 | 0.046 |
| LARC | NM_001102658 | chr15.71406839.71408839 | 1.642 | 0.046 |
| LARC | NM_003732 | chr5.139926250.139928250 | 1.642 | 0.046 |
| LARC | NM_004102 | chr1.31845135.31847135 | 1.644 | 0.046 |
| LARC | NM_152324 | chr13.111971990.111973990 | 1.646 | 0.046 |
| LARC | NM_018646 | chr7.142582490.142584490 | 1.667 | 0.046 |
| LARC | NM_001330417 | chr17.60004393.60006393 | 1.67 | 0.046 |
| LARC | NM_005961 | chr11.1035706.1037706 | 1.695 | 0.046 |
| LARC | NM_213600 | chr15.42447839.42449839 | 1.695 | 0.046 |
| LARC | NM_022482 | chr20.23341768.23343768 | 1.696 | 0.046 |
| LARC | NM_001271765 | chr17.73082821.73084821 | 1.711 | 0.046 |
| LARC | NM_012476 | chr2.71126719.71128719 | 1.713 | 0.046 |
| LARC | NM_001308269 | chr19.9694209.9696209 | 1.715 | 0.046 |
| LARC | NM_001282934 | chr21.43298591.43300591 | 1.717 | 0.046 |
| LARC | NM_153456 | chr13.96742092.96744092 | 1.722 | 0.046 |
| LARC | NM_001286259 | chr6.143762814.143764814 | 1.726 | 0.046 |
| LARC | NM_138435 | chr22.40389952.40391952 | 1.731 | 0.046 |
| LARC | NM_001242339 | chr10.3109818.3111818 | 1.736 | 0.046 |
| LARC | NM_001130088 | chr4.8159559.8161559 | 1.742 | 0.046 |
| LARC | NM_001300800 | chr11.63705802.63707802 | 1.743 | 0.046 |
| LARC | NM_176880 | chr19.19313238.19315238 | 1.745 | 0.046 |
| LARC | NM_001260508 | chr2.155554092.155556092 | 1.749 | 0.046 |
| LARC | NM_005778 | chr3.50125340.50127340 | 1.754 | 0.046 |
| LARC | NM_013278 | chr16.88704000.88706000 | 1.76 | 0.046 |
| LARC | NM_001278307 | chr2.238874586.238876586 | 1.769 | 0.046 |
| LARC | NM_013237 | chr5.176729762.176731762 | 1.775 | 0.046 |
| LARC | NM_001286688 | chr4.8072743.8074743 | 1.778 | 0.046 |
| LARC | NM_198501 | chr17.4486275.4488275 | 1.781 | 0.046 |
| LARC | NM_006591 | chr11.74302574.74304574 | 1.781 | 0.046 |
| LARC | NM_001317997 | chr9.86570901.86572901 | 1.785 | 0.046 |
| LARC | NM_033449 | chr5.141029986.141031986 | 1.787 | 0.046 |
| LARC | NM_001123392 | chr17.34756007.34758007 | 1.787 | 0.046 |
| LARC | NM_001080843 | chr22.24321338.24323338 | 1.791 | 0.046 |
| LARC | NM_001145725 | chr4.4290748.4292748 | 1.815 | 0.046 |
| LARC | NM_001291462 | chr17.34755993.34757993 | 1.824 | 0.046 |
| LARC | NM_001304369 | chr20.56802709.56804709 | 1.847 | 0.046 |
| LARC | NM_006255 | chr14.61787160.61789160 | 1.858 | 0.046 |
| LARC | NM_002036 | chr1.159172802.159174802 | 1.874 | 0.046 |
| LARC | NM_001315533 | chr21.44072861.44074861 | 1.897 | 0.046 |
| LARC | NM_014001 | chr17.73256742.73258742 | 1.899 | 0.046 |
| LARC | NM_015971 | chr17.73256748.73258748 | 1.899 | 0.046 |
| LARC | NM_175876 | chr1.231472618.231474618 | 1.923 | 0.046 |
| LARC | NM_001136140 | chr1.47798468.47800468 | 1.938 | 0.046 |
| LARC | NM_172231 | chr19.19430321.19432321 | 1.952 | 0.046 |
| LARC | NM_015508 | chr3.156391204.156393204 | 1.953 | 0.046 |
| LARC | NM_006645 | chr11.72503750.72505750 | 1.953 | 0.046 |
| LARC | NM_001166694 | chr15.78912637.78914637 | 1.958 | 0.046 |
| LARC | NM_001353010 | chr15.77196611.77198611 | 1.999 | 0.046 |
| LARC | NM_014585 | chr2.190444537.190446537 | 2.005 | 0.046 |
| LARC | NM_001267040 | chr6.15248085.15250085 | 2.008 | 0.046 |
| LARC | NM_030809 | chr12.51476454.51478454 | 2.038 | 0.046 |
| LARC | NM_007026 | chr17.35848950.35850950 | 2.05 | 0.046 |
| LARC | NM_001303428 | chr1.46597467.46599467 | 2.062 | 0.046 |
| LARC | NM_181521 | chr16.66729610.66731610 | 2.068 | 0.046 |
| LARC | NM_002478 | chr11.17740109.17742109 | 2.099 | 0.046 |
| LARC | NM_031890 | chr22.17601213.17603213 | 2.101 | 0.046 |
| LARC | NM_001163079 | chr22.17601257.17603257 | 2.101 | 0.046 |
| LARC | NM_001177639 | chr3.49506564.49508564 | 2.106 | 0.046 |
| LARC | NM_004204 | chr16.618967.620967 | 2.106 | 0.046 |
| LARC | NM_001409 | chr1.3527059.3529059 | 2.108 | 0.046 |
| LARC | NM_001568 | chr8.109259959.109261959 | 2.132 | 0.046 |
| LARC | NM_001297721 | chr1.154192273.154194273 | 2.133 | 0.046 |
| LARC | NM_001001890 | chr21.36259987.36261987 | 2.158 | 0.046 |
| LARC | NM_001316320 | chr1.11993723.11995723 | 2.16 | 0.046 |
| LARC | NM_006160 | chr17.37763175.37765175 | 2.174 | 0.046 |
| LARC | NM_001304537 | chr2.222436078.222438078 | 2.185 | 0.046 |
| LARC | NM_001168344 | chr6.7106829.7108829 | 2.206 | 0.046 |
| LARC | NM_001352510 | chr21.46961385.46963385 | 2.251 | 0.046 |
| LARC | NM_001003700 | chr6.7107085.7109085 | 2.286 | 0.046 |
| LARC | NM_033480 | chr6.52929256.52931256 | 2.288 | 0.046 |
| LARC | NM_001256295 | chr21.40176230.40178230 | 2.321 | 0.046 |
| LARC | NM_001184717 | chr3.156391714.156393714 | 2.335 | 0.046 |
| LARC | NM_006341 | chr1.11740271.11742271 | 2.364 | 0.046 |
| LARC | NM_001165031 | chr2.242625383.242627383 | 2.381 | 0.046 |
| LARC | NM_001286173 | chr12.22696480.22698480 | 2.586 | 0.046 |
| LARC | NM_001306089 | chr18.74533562.74535562 | 2.613 | 0.046 |
| LARC | NM_001353242 | chr8.131027943.131029943 | 2.726 | 0.046 |
| LARC | NM_001244008 | chr2.241758725.241760725 | 2.754 | 0.046 |
| LARC | NM_001890 | chr4.70795798.70797798 | 0.497 | 0.047 |
| LARC | NM_007051 | chr1.51424936.51426936 | 0.363 | 0.049 |
| LARC | NM_001080436 | chr19.34971879.34973879 | 0.446 | 0.049 |
| LARC | NM_001164766 | chr16.73091534.73093534 | 0.469 | 0.049 |
| LARC | NM_001039361 | chr1.13408127.13410127 | 0.496 | 0.049 |
| LARC | NM_001291381 | chr1.13408134.13410134 | 0.496 | 0.049 |
| LARC | NM_001145294 | chr3.25830530.25832530 | 0.531 | 0.049 |
| LARC | NM_006669 | chr19.55127383.55129383 | 0.532 | 0.049 |
| LARC | NM_213597 | chr17.8273858.8275858 | 0.549 | 0.049 |
| LARC | NM_001163941 | chr7.20654244.20656244 | 0.551 | 0.049 |
| LARC | NM_001193329 | chr9.97520930.97522930 | 0.554 | 0.049 |
| LARC | NM_001024630 | chr6.45295053.45297053 | 0.571 | 0.049 |
| LARC | NM_001190972 | chr8.91996485.91998485 | 0.575 | 0.049 |
| LARC | NM_000387 | chr3.48935426.48937426 | 0.6 | 0.049 |
| LARC | NM_003967 | chr6.132909877.132911877 | 0.612 | 0.049 |
| LARC | NM_014444 | chr15.43662256.43664256 | 0.645 | 0.049 |
| LARC | NM_001004471 | chr11.57995390.57997390 | 0.656 | 0.049 |
| LARC | NM_152459 | chr16.5115146.5117146 | 1.515 | 0.049 |
| LARC | NM_001128160 | chr3.33480870.33482870 | 1.717 | 0.049 |
| LARC | NM_024060 | chr11.62313332.62315332 | 1.725 | 0.049 |
| LARC | NM_001270601 | chr1.201083500.201085500 | 1.769 | 0.049 |
| LARC | NM_054113 | chr19.16283336.16285336 | 1.807 | 0.049 |
| LARC | NM_001301828 | chr10.135170529.135172529 | 1.819 | 0.049 |
| LARC | NM_020782 | chr12.27932186.27934186 | 2.08 | 0.049 |
| LARC | NM_182974 | chr9.138530386.138532386 | 2.168 | 0.049 |

FDR=false positive rate. LARC=locally advanced rectal cancer.

**Supplementary Table 2. Clinical characteristics of locally advanced rectal cancer**

|  | Training stage (n) | | |  | Validation stage (n) | | |
| --- | --- | --- | --- | --- | --- | --- | --- |
|  | Patients (135) | pCR (32) | Non-pCR (103) |  | Patients(59) | pCR (15) | Non-pCR (44) |
| Age | | | | | | | |
| <55 | 62 | 16 (26%) | 46 (74%) |  | 25 | 6 (24%) | 19 (76%) |
| >55 | 73 | 16 (22%) | 57 (78%) |  | 34 | 9 (26%) | 25 (74%) |
| Gender | | | | | | | |
| Male | 89 | 20 (22%) | 69 (78%) |  | 31 | 9 (29%) | 22 (71%) |
| Female | 46 | 12 (26%) | 34 (74%) |  | 28 | 6 (21%) | 22 (79%) |
| T stage | | | | | | | |
| T2 | 3 | 0 (0%) | 3 (100%) |  | 1 | 0 (0%) | 1 (100%) |
| T3 | 76 | 21 (28%) | 55 (72%) |  | 37 | 8 (22%) | 29 (78%) |
| T4 | 56 | 11 (20%) | 45 (80%) |  | 21 | 7 (33%) | 14 (67%) |
| N stage | | | | | | | |
| N0 | 23 | 7 (30%) | 16 (70%) |  | 12 | 4 (33%) | 8 (67%) |
| N1 | 61 | 14 (31%) | 47 (69%) |  | 23 | 4 (17%) | 19 (83%) |
| N2 | 51 | 11 (22%) | 40 (78%) |  | 24 | 7 (29%) | 17 (71%) |
| TNM stage | | | | | | | |
| II | 22 | 8 (36%) | 14 (64%) |  | 11 | 3 (27%) | 8 (79%) |
| III | 113 | 24 (21%) | 89 (79%) |  | 47 | 12 (26%) | 36 (74%) |

pCR=pathological complete response. TNM=tumour-node-metastasis.

**Supplementary Table 3. Thresholds of promoter profiling for each gene in PPCET**

| RefSeq | Symbol | Threshold |
| --- | --- | --- |
| NM_001303615 | MRGPRX2 | 4.91E-05 |
| NM_001004460 | OR10A2 | 5.22E-05 |
| NM_198447 | GOLT1A | 6.13E-05 |
| NM_198989 | DLEU7 | 5.63E-05 |
| NM_001286725 | KLHL1 | 5.19E-05 |
| NM_030973 | MED25 | 2.37E-05 |
| NM_005001 | NDUFA7 | 3.71E-05 |
| NM_001130069 | SUMF2 | 2.09E-05 |
| NM_001304509 | HDHD3 | 2.29E-05 |

Threshold means the thresholds for discretizing the continuous variables to one or zero.

**Reference**

1. Duffaud, F. & Therasse, P. [New guidelines to evaluate the response to treatment in solid tumors]. *Bull Cancer*. 87, 881-886 (2000).

2. Li, H.*, et al*. The Sequence Alignment/Map format and SAMtools. *Bioinformatics*. 25, 2078-2079 (2009).

3. An integrated encyclopedia of DNA elements in the human genome. *Nature*. 489, 57-74 (2012).

4. Casper, J.*, et al*. The UCSC Genome Browser database: 2018 update. *Nucleic Acids Res*. 46, D762-D769 (2018).

5. Chen, K.*, et al*. DANPOS: dynamic analysis of nucleosome position and occupancy by sequencing. *Genome Res*. 23, 341-351 (2013).

6. Quinlan, A.R. & Hall, I.M. BEDTools: a flexible suite of utilities for comparing genomic features. *Bioinformatics*. 26, 841-842 (2010).

7. Lin, X.J.*, et al*. A serum microRNA classifier for early detection of hepatocellular carcinoma: a multicentre, retrospective, longitudinal biomarker identification study with a nested case-control study. *Lancet Oncol*. 16, 804-815 (2015).

8. Robin, X.*, et al*. pROC: an open-source package for R and S+ to analyze and compare ROC curves. *BMC Bioinformatics*. 12, 77 (2011).

9. Zhou, Y.*, et al*. Metascape provides a biologist-oriented resource for the analysis of systems-level datasets. *Nat Commun*. 10, 1523 (2019).
